# Strategic choices of migrants and smugglers in the Central Mediterranean sea

**DOI:** 10.1371/journal.pone.0300553

**Published:** 2024-04-19

**Authors:** Katherine Hoffmann Pham, Junpei Komiyama

**Affiliations:** Department of Technology, Operations, and Statistics, NYU Stern School of Business, New York, NY, United States of America; University of Macerata, ITALY

## Abstract

The sea crossing from Libya to Italy is one of the world’s most dangerous and politically contentious migration routes, and yet over half a million people have attempted the crossing since 2014. Leveraging data on aggregate migration flows and individual migration incidents, we estimate how migrants and smugglers have reacted to changes in the border enforcement regime, namely the rise in interceptions by the Libyan Coast Guard starting in 2017 and the corresponding decrease in the probability of rescue to Europe. We find support for a deterrence effect in which attempted crossings along the Central Mediterranean route declined, and a diversion effect in which some migrants substituted to the Western Mediterranean route. At the same time, smugglers adapted their tactics. Using a strategic model of the smuggler’s choice of boat size, we estimate how smugglers trade off between the short-run payoffs to launching overcrowded boats and the long-run costs of making less successful crossing attempts under different levels of enforcement. Taken together, these analyses shed light on how the integration of incident- and flow-level datasets can inform ongoing migration policy debates and identify potential consequences of changing enforcement regimes.

## Introduction

There are approximately 272 million international migrants around the world [1], and estimates suggested that a quarter of the international migrant stock were irregular migrants as of 2009 [2]. Smuggling networks often enable such flows, moving an estimated 2.5 million people in 2016 for an annual profit of $5.5–7 billion [3]. Limiting human smuggling may be desirable from a security perspective (e.g., in order to enforce the sovereignty of borders) or from a humanitarian perspective (e.g., to reduce exploitation and trafficking of undocumented people and discourage migrants from risking their lives on dangerous crossings). However, past research has found that efforts to limit human smuggling by increasing border enforcement may lead to unintended consequences. For example, researchers have identified a “deterrence-diversion tradeoff” in which some migrants forego the journey altogether, but others adapt by switching to alternative routes which may place them at higher risk [4]. As a result, it is difficult to estimate the effects of migration policies, and there is a need for system-level models to estimate how migrants and smugglers react to changes in the crossing environment.

In this paper, we aim to build such models and apply them to study sea crossings on the Central Mediterranean route from Libya to Italy. Since 2014, over two million people have arrived in Europe by sea and over 20,000 people have died or gone missing [5]. There are three primary routes through the Mediterranean, illustrated in Fig 1: the Eastern route from Turkey to Greece (approximately 58% of crossings from 2014 to 2020), the Central route from Libya to Italy and Malta (approximately 34%), and the Western route from Morocco to Spain (approximately 8%) [5]. We focus on the Central Mediterranean route, which is the longest of the three and represents approximately 81% of all casualties observed in this period [6]. In recent years, this route has been at the center of a contentious policy debate about the role of non-governmental organization-led (NGO-led) rescues and coast guard interceptions in encouraging or deterring risky migration attempts by sea.

**Fig 1:**
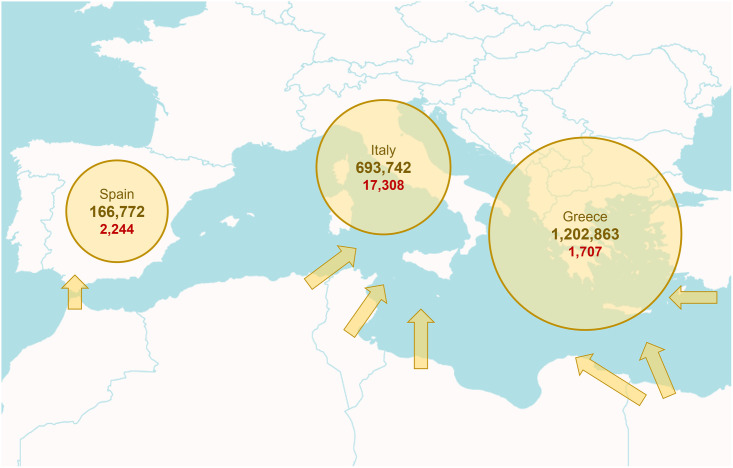
Flows along the Western, Central, and Eastern Mediterranean routes from 2014–2020. The gold number shows the total number of arrivals to the primary destination country for each route [5]. The red number below shows the number of dead or missing migrants along the route [6]. Graphic adapted from [5].

While a number of researchers have attempted to model adaptive responses to migration conditions in the Central Mediterranean [7–11], much of this work focuses on analyzing the rise in NGO rescues and the surge in crossings from 2015–2017, when arrivals to Italy by sea peaked at over 27,000 people in a given month [12]. There has been less systematic analysis of the region after mid-2017, when the Libyan Coast Guard (LCG) began to intercept a growing share of migrants with Italian and European Union (EU) support, and information on the activity of migrants and smugglers became harder to obtain due to the LCG’s lack of consistent and detailed reporting. In the face of the drastic change of environment caused by the increased role of the LCG, we seek to answer the following policy questions.

1. *To what extent does the shift toward Libyan interceptions, and away from EU or NGO-led rescues, deter the crossing of migrants?* Do increased interceptions on the Central route divert migrants towards other migration routes? Is there any causal relationship that we can identify from the data?
2. *How do smugglers, who help the migrants cross the sea, attempt to ensure success in this new enforcement environment?* Is there any evidence around how they adapt their strategy at the incident level, and what are the outcomes of these strategy adaptations?

Answering these questions is significantly more challenging than in previous periods due to the scarcity and bias of incident datasets after the increase in Libyan interceptions in mid-2017. In particular, compared with the reports produced by NGOs which are motivated to disclose their rescue incidents, details on interceptions by the LCG (as well as casualties that occur as a consequence of LCG activities) are unpublished and the number of interceptions is reported only in aggregate. The most comprehensive dataset from the EU border control agency (Frontex) does not generally cover boats intercepted by the LCG at all. Therefore, we have a combination of biased but high-quality incident data, and comprehensive but low-quality flow data. While this type of data is common in many informal migration settings (where interceptions are often performed by non-transparent enforcement authorities), it is difficult to analyze because many of the conventional causal analysis methods do not apply to the situation. Indeed, few researchers have analyzed incident-level data on crossings, limiting their ability to study the evolution of smuggler strategy at the boat level. While [8, 11] do use incident data, they aggregate it at the daily level for their analysis.

Our main contribution is therefore the causal analysis of Central Mediterranean crossing behavior both before and after the rise in Libyan interceptions, with careful robustness checks. Unlike existing papers that focus on the causal impact of NGO presence [8, 9] or civil disorder and discontinuous policy change [11], we focus directly on the rate of successful crossing, which determines the benefit that migrants expect to obtain by their crossing attempts. We integrate the flow and the incident data by obtaining a reasonable estimate of the overall rate of interceptions across time, which we then associate with each recorded crossing attempt. We confirm that the probability of successful crossing affects multiple aspects of the Central Mediterranean crossing, from the volume of crossing attempts to crossing characteristics (namely, the boat size).

We conduct two primary analyses. First, we build a time series model to estimate how the flow of crossings on the Central Mediterranean route responds to the growing rate of LCG interceptions and the decline in NGO and EU-led rescue activity off the Libyan coast, which increases the proportion of people returned to Libya and reduces migrants’ probability of successful rescue to Europe. To set the stage, we fit a gravity model on data from all three routes to estimate the flow of migrants between countries based on the rescue probability, push factors in the country of origin, and country-pair factors such as distance and diaspora size. Across several different specifications, we demonstrate that the rescue probability has a meaningful, statistically significant positive association with the number of crossings. To avoid concerns about finding false causal relationships due to spurious correlations, we next focus on rescues on the Central Mediterranean route and estimate an Error Correction Model (ECM) for this setting. We find a long-run positive relationship between rescue probability and attempted crossings. This is consistent with the somewhat surprising resurgence of sea migrants observed in the third quarter of 2019, as the probability of Libyan interception declined. The ECM’s second-stage model of the number of crossings has an explanatory power of *R*^2^ = 0.20 (adjusted *R*^2^ = 0.16), which implies that the probability of successful rescue to Europe has a meaningful effect on the flow size. We estimate that a decline in rescue probability from 90% to 50% corresponds to over 10,000 fewer attempted monthly crossings on average, from over 14,600 expected crossings to approximately 3,400 crossings.

Second, we analyze incident data to document the strategic response of smugglers to the increased probability of interception and the reduction in EU and NGO-led rescues off the Libyan coast. As LCG interceptions rise, anecdotal and observational evidence suggests that smugglers and migrants begin to prepare for longer voyages towards Europe. They increase the use of wooden boats relative to rubber rafts, and reduce the average number of people on board. To systematically explore crossing strategy, we construct a theoretical model of smuggler utility as a function of boat size. We adopt a random utility maximization model that connects the flow-level data (the rate of interception) and the incident-level data (boat size) and enables counterfactual estimation with a limited amount of incident records. We estimate the strategic tradeoff between the short-run incentive to crowd more passengers onto rubber boats, and the long-run incentive to avoid Libyan interception and reach the EU Search and Rescue (SAR) zones by using smaller boats. For rubber boats, we estimate that smaller boats (≤ 50 people) begin to dominate larger boats (> 100 people) as the preferred alternative once the rate of Libyan interception approaches 60%. These results are consistent with the rise in the use of smaller boats in early 2019, when the intensity of LCG activity peaked. This implies that our model can capture continuous changes in the enforcement situation, unlike before-after discontinuity models.

In summary, we build on the evidence base for evaluating policy changes in the Central Mediterranean. The policy implications of our results are as follows:

- Our analysis of the flow-level dataset is consistent with the claim that the shift towards LCG interceptions, and away from EU or NGO-led rescues, decreases the volume of crossings. We find that this adjustment occurs relatively quickly in response to changes in the rate of interception. However, we note that the crossings which *do* continue despite increased enforcement are likely more perilous because it is less likely that distressed boats will be met with a rescue response, due to reduced rescue capacity in the region and to the fact that they might actively avoid seeking help from the LCG.
- Our analysis finds evidence of a constrained diversion effect in which some migrants switch to the Western Mediterranean route when the chance of successful crossing on the Central route is low. We note that our estimate of the diversion effect is likely to be an underestimate, since we focus on quantifying shifts *across* routes rather than shifts *within* the Central Mediterranean route. Descriptive analysis suggests that the extent of substitution varies by nationality and likely depends on the ease with which migrants can reach coastal departure points from their respective countries. This suggests that rather than focusing on conducting interceptions at individual crossing points (which can simply push migrants towards other crossing points that are less well policed and potentially more treacherous), policymakers should take a comprehensive view of smuggling routes more generally. This is consistent with broader calls for integrated regional approaches to addressing the underlying drivers of migration, and for the expansion of safe and legal crossing routes [13].
- Our analysis of incident data suggests that smugglers adapt to the changes in the enforcement regime, and migrants still succeed in securing rescues to Europe in the current environment. Even at times when the LCG operated most actively to block migrants transiting the Libyan coastal zone, its effectiveness may have been limited by smugglers’ strategic response. Boats with smaller numbers on board are estimated to have had an advantage in passing the coastal region and reaching European search and rescue zones, and our model makes it possible to estimate how smugglers weigh this advantage against the possibility of collecting more profit by adding passengers. Conditional on rescue to Europe, our analysis suggests that the risk of death for passengers has not changed from one period to the next, despite this shift in strategy. However, as smugglers switch to smaller boats which are rescued farther out to sea, it is possible that there is an increasing number of boats which sink without ever being detected for a rescue attempt, thus biasing recent casualty estimates downwards.

The rest of the paper is structured as follows. We begin with contextual information on the Central Mediterranean policy environment and a review of the existing literature on migration strategy. We continue with an analysis of the overall flow of crossings along the Central Mediterranean route. Then, we turn to an analysis of individual crossing incidents and present a utility model of smuggling. The final section concludes with a discussion of implications and future work.

### Background

The difficulty of policing maritime borders has long made the Mediterranean an attractive route into the European Union. In particular, the Central Mediterranean route from Libya to Italy and Malta draws migrants and refugees who have lived and worked in Libya for years, as well as those who use Libya as a transit country. All together, since 2014 UNHCR reports that over 690,000 people have attempted the Central Mediterranean sea crossing [12] and over 56,000 have been returned to Libya [14], while the International Organization for Migration (IOM) estimates that over 17,000 people have gone dead or missing along this route [6].

Given the risks involved in the crossing, European policymakers are divided between the humanitarian imperative to save lives in the Central Mediterranean through search and rescue, and the desire to stop irregular migration flows and discourage risky migration. On the one hand, migrants have legitimate reasons for fleeing Libya, where they have faced discrimination, human trafficking, detention in inhumane conditions, and the risk of air strikes from the civil war [15, 16]. Conditional on successfully departing from Libya, they should be protected from forcible return, since international law requires that rescued migrants, refugees, and asylum-seekers be transported to a “place of safety” [17]. On the other hand, smugglers are aware of these protections and actively manipulate them by sending migrants to sea in under-equipped boats that will require rescue. As crossings surged in 2016 the Italian authorities were reporting as many as 30 rescue operations a day, leading to accusations that search and rescue operations were acting as a “ferry service” for migrants and creating a “pull factor” which encouraged people to place their lives at risk [18, 19].

#### Policy context

Below, we characterize the recent policy response in the Central Mediterranean according to three main phases: the dominance of Italian and EU naval missions; the rise of the NGO rescue response; and the growing role of the Libyan Coast Guard. We describe the phases in terms of the activities of these key actors, and thus some parts of them are overlapping.

*Phase 1: The dominance of Italian and European naval missions (up to mid-2015)*. Large-scale search and rescue operations off the coast of Libya began as early as 2013, when the Italian Navy launched the one-year-long Operation Mare Nostrum which assisted over 150,000 individuals [20]. At the end of 2014, Mare Nostrum was replaced by the joint EU Operation Triton. While Mare Nostrum was explicitly a life-saving operation, Triton emphasized border control and initially restricted naval patrols to a smaller area of the sea [8]. The cost of this policy change became clear in April 2015 when two major shipwrecks claimed over 1,000 lives [21]. In response to these tragedies, the EU launched Operation Sophia in June 2015, which supplemented Triton with an emphasis on preventing smuggling and destroying migrant ships so they could not be re-used in the future [22]. At the same time, a number of NGO rescue ships began to operate in the region in order to further increase SAR capacity.

*Phase 2: The rise in NGO rescues (from mid-2015 to mid-2017)*. The first NGO to operate in the Mediterranean was the Migrant Offshore Aid Station (MOAS), which began conducting rescues in 2014. In the spring of 2015 it was joined by Médecins sans Frontières (MSF, also known as Doctors without Borders), and several other NGOs have since followed suit. When crossings peaked in 2016–2017 there were as many as 13 different boats operating in the region [23]. The NGO presence increased the coordination of rescues, as NGOs would deploy off the coast of Libya and react quickly to any boats that were identified in international waters. However, this led to accusations that NGOs were colluding with smugglers and facilitating irregular migration.

In July 2017, Italy proposed an NGO Code of Conduct which would impose a number of constraints on rescue ships wishing to use its ports [24, 25]. European countries also began other efforts to limit the operations of NGO ships, for example by initiating legal proceedings against them, preventing them from leaving port, or denying permission to disembark rescued migrants [26]. These efforts to restrict the activity of rescue NGOs coincided with the increasing delegation of rescue authority to the LCG.

*Phase 3: Interceptions by the Libyan Coast Guard (since mid-2017)*. Search and rescue activity is typically managed by an international network of coastal countries, which officially declare rescue zones and then supervise the response to incidents within their zones. Formally, the coastal zone within 12 nautical miles (NM) from a country’s shore is considered *territorial waters*, and from a legal perspective is essentially treated like the land within the country’s borders. Between 12 and 24 NM from shore are a country’s contiguous waters, a zone which is not technically part of the country’s territory but in which the country may still enforce some of its laws. Beyond this 24 NM boundary are the search and rescue (SAR) zones, in which the corresponding country’s coast guard and/or naval forces are responsible for coordinating rescue operations [27].

Key SAR zones in the Central Mediterranean are shown in Fig 2. Prior to the formal recognition of the Libyan SAR zone in 2018, the Maritime Rescue Coordination Center (MRCC) in Rome coordinated rescue efforts off the coast of Libya, and rescued migrants were typically taken to Europe. However, as of October 2016 the EU began training the LCG, and as of May 2017 MRCC Rome began assigning rescues to LCG boats [27]. This policy shift was formalized by the Malta Declaration in February 2017, in which the EU committed to support the training and capacity building of the LCG for the purpose of migration enforcement, and in June 2018, when Libya’s request to formally establish a SAR zone was approved [27, 28].

**Fig 2:**
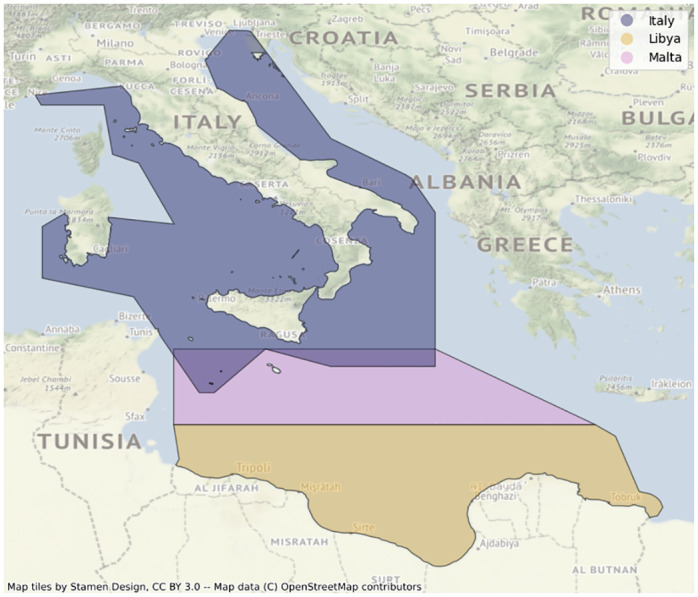
Map of Italian, Maltese, and Libyan search and rescue zones. The spatial extent of each country’s search and rescue zone was manually calculated by combining Natural Earth boundaries with the coordinates reported in the International Maritime Organization’s global search and rescue plan, updated in 2017 with Libya’s rescue zone [29–31].

As a consequence of these changes, a growing number of calls to the MRCC in Rome were passed to the LCG. In addition to reducing its naval SAR activities in the region, the EU began deploying aerial assets to monitor the Libyan SAR zone and request LCG intervention before migrant ships entered the Italian or Maltese SAR zones [32]. There is evidence that some migrants have even been “pushed back” to Libya after successfully reaching EU SAR zones [33].

The delegation of rescue responsibility to the LCG and the EU’s so-called “non-assistance” policies have been widely condemned by the human rights community [15, 27, 28, 34, 35]. While international ships are generally prevented from returning migrants to Libya because it is not considered a place of safety [36], the LCG does not abide by these restrictions, and the increasing interventions of the LCG have clearly imperiled migrants. There are reports that the LCG is poorly trained, unprofessional, and ill-equipped to coordinate rescues [37]; that it often cannot be reached by phone and/or is unresponsive in case of emergencies [38]; that it has failed to consistently pay its staff, who as a result may not report for duty until needed [39]; and that it has perpetrated abuses against migrants in the course of rescues [15, 40], including shooting at migrant boats outside of its rescue zone [41, 42]. Rather than acting as an independent and unified force, the LCG has ties to militias involved in Libya’s civil war [43, 44], and reports suggest that the LCG has diverted its resources to the war effort [45, 46].

#### Smuggling operations and strategy

The United Nations Office on Drugs and Crime (UNODC) estimates that almost all migrants who cross the Central Mediterranean rely on the help of smugglers [3]. Migrants pay smugglers for passage, with payment depending on the type of boat, the location of the migrant on the boat, the nationality of the migrant, and the month and year of departure. For example, in 2015 a report found that on the same vessel, Syrians in preferred locations might pay $2,500 while sub-Saharan Africans in the hold might pay just $800 [47]. By 2017, the price of crossing in a rubber boat had fallen to $90 or below [48].

Migrants typically depart at night, most commonly in a wooden fishing boat or a rubber raft. Since the EU-led anti-smuggling Operation Sophia began destroying vessels in 2015, the incentive to purchase cheaper disposable rafts has increased [49]. Wooden boats can hold up to 800 people [50], whereas rafts have a maximum capacity of approximately 150–200 people. When boats are overcrowded, the risk of sinking and injury to passengers on board is expected to rise. Therefore, smugglers face a trade-off between collecting additional revenue per passenger and the risks incurred by overloading the boats.

Migrant boats are optionally equipped with life vests and a satellite phone, and one of the migrants may be chosen to act as navigator. At the peak of the rescue response, boats were often given a limited amount of fuel, with the goal of reaching international waters [51]; once they passed the 12-nautical-mile boundary from the Libyan shore, they could use their satellite phone (if available) to request a rescue from the MRCC in Rome. As the LCG has grown more active in intercepting ships, anecdotal reports suggest that smugglers are equipping boats with more fuel in order to help them travel past the LCG and get further out to sea before requesting assistance [36]; the space taken by the fuel may in turn reduce the passenger capacity of the boats.

While the LCG is theoretically an adversary of the smuggling operations (since the coast guard is charged with intercepting migrant boats), there is evidence that LCG membership has included known migrant smugglers—even in leadership positions [52, 53]—and that LCG members coordinate with, profit from, and/or remain involved in smuggling operations [44], for example by accepting payments to allow certain migrant boats to pass [43, 54].

### Related work

Our study of Central Mediterranean crossings fits into the larger literature on human migration, which models movement patterns as a function of costs (such as travel expenses) and benefits (such as employment opportunities). Informal migration is differentiated by the presence of a third factor: internal and external border enforcement [55].

#### Strategic models of migrants and smugglers

Smuggling and trafficking involve sophisticated, diversified organizations: “the business is remarkably responsive to change and seems always to remain one or several steps ahead of those seeking to control it” [56]. Adaptation has been a key theme in existing research on the US-Mexican border, particularly with respect to the geographic intensity of US border patrol activities. For example, [4] posit that enforcement has two primary effects: (1) a *deterrence effect* in which the policy discourages migrant crossings; and (2) a *diversion effect* in which migrants shift their crossings to other parts of the border. As a result of the diversion effect, the overall volume of crossings can be relatively inelastic with respect to border enforcement. Additional empirical research has found a low impact of enforcement on overall crossing volumes, but substitution to other border sectors with higher crossing times and crossing risk and an increase in the relative proportion of deaths from environmental factors such as dehydration [57, 58].

#### Analyses of Central Mediterranean crossings before Phase 3

In the Central Mediterranean, the deterrence-diversion debate has been shaped by two competing narratives: a security/border control logic, and a humanitarian/crisis discourse [7]. A core research question is whether limiting rescue activity and increasing interceptions will discourage attempted crossings, or simply cause migrants to undertake increasingly risky crossings which are unassisted or even unobserved by state and humanitarian actors. To date, some studies have suggested that rescue presence does not increase migrant departures or deaths [7, 9].

[8] construct a theoretical model of how migrants and smugglers react to crossing risk in order to analyze behavioral responses to policy changes. In their model, migrants make a strategic decision about whether to cross in a safe boat, an unsafe boat, or not at all. They predict that SAR efforts lower crossing risks conditional on boat type, and therefore: encourage more migrants to undertake the journey; lead a larger fraction of crossings to use unsafe boats; and consequently, make departures more sensitive to crossing conditions.

[10] approach the problem from a different angle, building an inter-temporal matching model between migrants and smugglers. They predict that greater NGO presence in the Mediterranean will increase the number of migrants and smugglers, lower the costs of smuggling, and increase the likelihood of successful crossing. Smugglers will benefit but the effect on migrant welfare and crossing prices is ambiguous.

#### Analyses of Central Mediterranean crossings including Phase 3

Policy analysis of Central Mediterranean crossings given the recent rise in LCG intervention faces two key challenges. First, because of the changing political and economic environment, it can be difficult to isolate the causal effect of policy changes on crossing behavior. Prior works have studied the growing role of the LCG as a discontinuous policy change [11], and studied how crossings correlate with NGO presence or capacity on a daily basis [9]. However, there have been no studies that assess how continuous changes in the overall border enforcement regime affect crossings. We address this gap with the use of an error correction model which allows us to estimate how crossing decisions on the Central and Western Mediterranean routes respond to rescue probabilities in the short and long term. With the help of this model we are able to analyze recent crossing activity through the end of 2019, when we observe a recovering flow of migrants.

Second, the lack of data on events involving the Libyan Coast Guard makes it difficult to connect smuggler choices (i.e. departure ports, boat type, or boat capacity) to outcomes at the incident level, since there is almost no data on boats that are intercepted. This hinders efforts to identify smuggler strategy because it hides the smuggler’s reward function. To address this limitation, we borrow from random utility maximization models and their connection to inverse reinforcement learning [59–61]. Using data on the characteristics of a given incident and the choices made by the smugglers, we attempt to infer the parameters of the smuggler’s utility function. Specifically, we study the question of boat crowding, and estimate the value that smugglers place on the profit collected from adding more passengers, relative to the reduced chance of success when using larger boats (which in turn depends on the overall level of Libyan enforcement). We estimate this choice model with newly released Frontex data which, to our knowledge, has not been analyzed in its entirety to study this context.

#### Limitations of our research

Our approach has four key limitations. First, as noted above our flow data analysis focuses on shifts from the Central to the Western Mediterranean route, rather than shifts *within* the Central Mediterranean route. In our datasets, we found evidence suggesting that preferred ports of departure within Libya change over time, and that recent years have seen a growing share of migrant activity originating in Tunisia. Due to limitations in our flow dataset (namely, the fact that IOM reports arrivals and deaths disaggregated by route or arrival country, rather than by country of origin or departure port), we do not focus on these trends. However, since our analysis consequently does not quantify these within-route shifts, our estimate of the diversion effect is likely to be conservative.

Second, we assume that migrants are free to leave (though they may soon be intercepted at sea), and therefore we do not account for efforts to stop migrants from departing in the first place. [11] note that reductions in migrant flows during and after 2017 may be a function of either increased coast guard interceptions, or agreements with militias to reduce the availability of smuggling services and prevent departures in the first place; our analysis addresses the former.

Third, we make the simplifying assumption that migrant smugglers and the LCG are adversaries. As described above, this assumption may not be accurate, since there is evidence that members of the LCG accept bribes to allow some boats to pass, while targeting others for return. However, the extent of this phenomenon is difficult to quantify. Even where such schemes are in place, we note that there should be a connection between boat type choices and success. Migrant ships which do not have an agreement with the LCG should should still have an incentive to evade it, whereas the reduction in EU and NGO rescue capacity in the Libyan SAR zone means that boats which are allowed to pass must generally still successfully navigate to the European SAR zones. Thus boat type choices should be made based on the need to evade coast guard patrols and to travel long distances before rescue, both of which increase in periods when the rate of LCG interceptions is high.

Finally, while we believe that Frontex collects the most comprehensive incident data in the region, its sample of incidents is biased because it generally does not include Libyan interceptions. This may lead us to overestimate the extent to which smugglers are strategic (because non-strategic actions are more likely to be filtered out of the dataset by coast guard interceptions). However, LCG activities are erratic, with gaps in activity on certain days [37, 38], which should help to counteract this bias by ensuring that more “non-strategic” boats are able to evade detection and enter the Frontex dataset.

## Analysis of the aggregate flow dataset

A common argument in favor of stricter border enforcement is that a decreased chance of successful crossing will deter crossing attempts. In this section, we assess whether or not this hypothesis is consistent with empirical estimates by investigating how crossings relate to the likelihood of rescue at sea and successful arrival in Italy or Malta.

### Descriptive analysis

If deterrence and diversion effects are occurring as a result of rescue policy changes, we expect two conditions to hold. First, the volume of attempted crossings should vary over time (consistent with deterrence). Second, there must be variation in route choice (consistent with diversion). We begin with a descriptive analysis of crossings by route and nationality to investigate whether the data displays these characteristics, before exploring these questions more systematically with the use of gravity and error correction models.

#### Data

We collect data on overall migration flows from the International Organization for Migration (IOM), which provides data on aggregate sea arrivals to Italy, Malta, Spain, and Greece; interceptions by the Libyan, Tunisian, and Turkish Coast Guards (unfortunately, interception data from Morocco is unavailable); and dead or missing migrants along the Central, Western, and Eastern Mediterranean routes [62]. We assume that the number of sea arrivals is essentially equivalent to the number of people rescued because very few boats reach Europe independently.

We use this data to calculate the total number of crossing attempts and the likelihood of successful crossing. For a given month *t*, we define the number of people crossing on a given route as the sum of people who were rescued, intercepted, or reported dead or missing:

$$N_{t,cross}=N_{t,rescue}+N_{t,intercept}+N_{t,death}.$$

The probability of rescue is therefore:

$$P_{t,rescue}\;=\;\frac{N_{t,rescue}}{N_{t,cross}}.$$

Note that there is unavoidable measurement error in the calculation of *N*_*t*,*cross*_ as we do not have data on the number of people who have entered Europe undetected or the number of people who went missing at sea without a trace; as noted above, we are also missing interception data for the Western route (i.e., Morocco). While we believe that this is the best available estimate of crossings, due to these data gaps, this term is almost certainly an underestimate.

To complement this route-level data, we also gather information on country-level flows from UNHCR, which reports data on monthly arrivals to Italy, Greece, and Spain from 2016–2019, broken down by country of origin [5, 63]. Unfortunately, this dataset does not distinguish between land and sea arrivals at the level of the origin country, so all arrivals are analyzed together when using this data.

#### Results

The number of people crossing on the Central Mediterranean route, as well as the probability of each outcome (rescue, interception, and going missing/death), are shown in Fig 3. From inspecting the figure, it is clear that the number of people crossing and the probability of rescue have fallen over time, which coincides with the growing intervention by the LCG in Phase 3. However, it is unknown whether both series simply follow a common downward trend, or whether one series reflects changes in the other over the short or long term. We investigate this causal question in more detail below in the “Error correction model” section.

**Fig 3:**
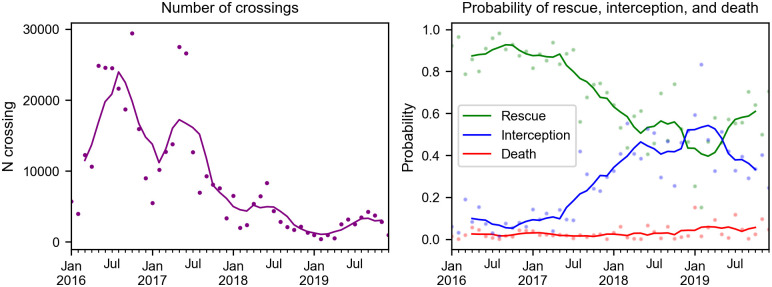
Crossing decisions and crossing outcomes on the Central Mediterranean route over time. Each dot represents the monthly number of crossings or the monthly probability of the respective outcome, whereas the lines represent centered six-month moving average trends. Data source: [62].

Figs 4 to 6 illustrate the share of African migrants arriving on each route by nationality over time. We can see that the number of departures varies within and across countries. Furthermore, the country-level flows are consistent with substitution over routes. While North and East Africans generally take the Central Mediterranean route (with the exception of Moroccans and Algerians, who are proximate to Spain), West Africans have generally switched from the Central Mediterranean route to the Western Mediterranean route over time. This is likely due to the fact that from West Africa, multiple overland routes exist to either Western or Central Mediterranean departure points, and travel is facilitated by freedom of movement across borders within the 15 members of the Economic Community of West African States (ECOWAS) [64].

**Fig 4:**
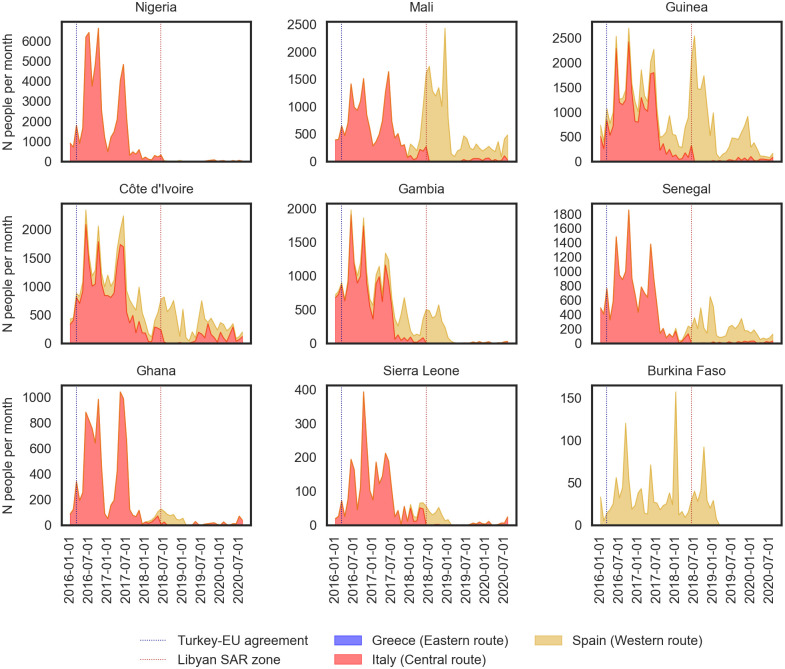
The number of migrants crossing by nationality and route: West Africa. The following West African countries had less than 150 monthly arrivals in all time periods (all via the Spanish route) and were omitted from the plots: Mauritania, Guinea-Bissau, Togo, and Liberia. Data source: [63].

**Fig 5:**
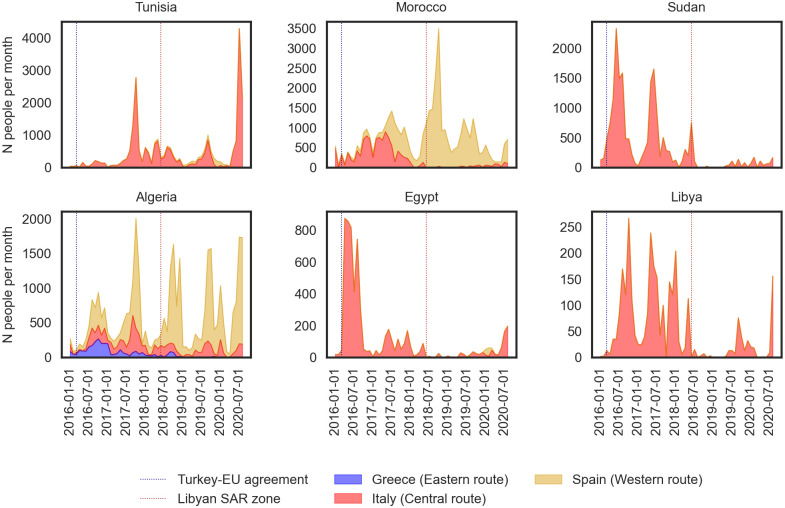
The number of migrants crossing by nationality and route: North Africa. Data source: [63].

**Fig 6:**
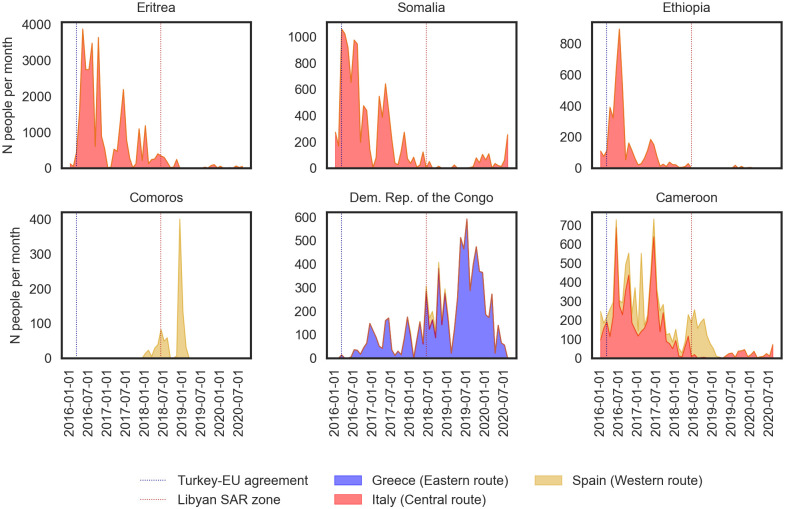
The number of migrants crossing by nationality and route: East and Central Africa. The following Central African countries had less than 50 monthly arrivals in all time periods (all via the Spanish route) and were omitted: the Republic of the Congo, the Central African Republic, and Chad. Data source: [63].

Taken together, we can see that many nationalities’ preference of migration route varies over time, and this variation seems to coincide with major policy changes, such as the growing role of the LCG in intercepting migrants as formalized by the establishment of the SAR zone in June 2018. However, the volume of arrivals, the routes chosen, and the sensitivity of this choice to crossing conditions vary substantially by region of origin.

### Gravity model

To better understand these trends, we next fit a gravity model to explain the number of arrivals according to various country and country-pair characteristics. The gravity model is a common approach to modeling migration that typically estimates pairwise flows between countries as a function of push factors that drive migrants from their place of origin, pull factors that attract them to their destination, and pairwise factors (such as distance) that may influence the cost of migration [65].

#### Data and setup

In order to capture pairwise migration flows, we use the UNHCR dataset described above [63], which reports the monthly number of arrivals from one of 37 origin countries, recorded in one of the three destination countries (Spain, Italy, or Greece).

Due to the limited number of destination countries analyzed, and because the country of arrival in the EU may not be migrants’ final destination country, our analysis focuses on push and pairwise factors. The key independent variable of interest is the monthly rescue probability on the relevant (Eastern, Central, or Western) route according to IOM data, constructed as described above in the section on Descriptive analysis. In addition, we include data on factors including: the pairwise distance between countries, collected from the US International Trade Commission’s dynamic gravity dataset [66]; twelve indicators from the Fund for Peace’s Fragile States Index, which capture social cohesion, economic factors, political factors, and social factors at an annual level in the source countries [67]; and the total number of people born in the source country who are living in the destination country, collected from Eurostat [68]. Additional details on the gravity dataset can be found in S2 Appendix in S1 File.

The final dataset includes 48 monthly observations for each of 58 origin-destination country pairs. However, Eurostat diaspora data is missing for several country pairs whose destination is Spain and for all country pairs with destination Greece, leaving only 32 country pairs with complete records in the dataset.

#### Model

Above, we describe how the number of attempted crossings at the *route* level can be calculated by adding together arrivals, interceptions, and the number of people dead and missing at sea. However, at the *country pair* level, we have only the total number of arrivals by land and sea.

To infer the total number of attempted crossings at the country pair level, we first treat all UNHCR-reported arrivals as sea crossings (as mentioned above, the UNHCR data does not report land and sea arrivals separately for each country of origin and month). Then, we scale the number of arrivals for each country pair using the route-level rescue probability. Specifically, we calculate the number of attempted crossings from origin *o* to destination *d* in month *t* as:

$$N_{cross,t}^{o,d}=\frac{N_{arrivals,t}^{o,d}}{P_{rescue,t}^{route}}$$

where $N_{arrivals,t}^{o,d}$ is the monthly number of reported arrivals from the origin country to the destination country, and $P_{rescue,t}^{route}$ is the probability of rescue along the route. This assumes that all nationalities have an equal probability of rescue when using the same route in a given month, which is plausible if they are crossing from the same departure points on the same vessels.

Given this monthly number of attempted crossings, we next estimate a gravity model of the form:

$$\begin{array}{ccc} N_{cross,t}^{o,d} & = & \text{exp}(\;\beta_{1}P_{rescue,t-1}^{route}\;+\;\beta_{2}FSI_{t}^{o}\;+\;\beta_{3}\;\text{log}\left(Distance^{o,d}\right)\\ & & \;+\;\beta_{3}\;\text{log}\left(Diaspora_{t-12}^{o,d}\right)\;+\;\alpha_{t}\;+\;\gamma_{d}\;+\;\eta_{t}^{o,d}\;) \end{array}$$

where $FSI_{t}^{o}$ are the indicators from the Fragile States Index; *Distance*^*o*,*d*^ is the pairwise distance between countries; $Diaspora_{t-12}^{o,d}$ is the number of people born in the origin country who reside in the destination country (i.e., the migrant stock); *α*_*t*_ are a set of month fixed effects; *γ*_*d*_ are a set of destination fixed effects; and $\eta_{t}^{o,d}$ is an error term that is assumed to be clustered at the level of the origin-destination pair. Since the rescue probability $P_{rescue,t}^{route}$ is used in the calculation of the dependent variable ($N_{cross}^{o,d}$), we lag it by one month before including it as an independent variable. Similarly, because the annually-measured diaspora variable $Diaspora_{t}^{o,d}$ may be a direct function of the number of crossings, we lag it by twelve months before including it as an independent variable.

We estimate this model using Poisson Pseudo-Maximum Likelihood estimation [69], as implemented by the ppmlhdfe command in Stata 18.0 [70].

#### Results

Results from estimating the gravity model are shown in Table 1. Our preferred specification is in column (2). However, we also present specifications without the diaspora variable (columns (1) and (3)) because including this variable significantly decreases the dataset size (observations from Greece and Spain are removed where the diaspora variable is missing, as described above). Furthermore, we test specifications (columns (3) and (4)) in which destination fixed effects *γ*_*d*_ are replaced by origin-destination fixed effects, which complicates interpretation of the coefficients on the Fragile States Index variables and the diaspora variable, but appears to capture more of the variation between country pairs and can help control for frictions in movement between countries that are not explicitly captured by other independent variables in our model.

**Table 1:** Results of estimating the gravity model: Factors influencing attempted crossings between country pairs.

	$N_{cross,t}^{o,d}$			
	(1)	(2)	(3)	(4)
$P_{rescue,t-1}^{route}$	6.271***	6.501***	6.206***	5.404***
	(0.774)	(1.159)	(0.690)	(0.848)
FSI: Security Apparatus	0.163	0.342**	0.231	-0.739*
	(0.136)	(0.162)	(0.282)	(0.422)
FSI: Factionalized Elites	-0.164	-0.283***	-0.275	1.114
	(0.124)	(0.109)	(0.348)	(0.832)
FSI: Group Grievance	0.165	-0.128	-0.883***	-0.245
	(0.119)	(0.236)	(0.299)	(0.382)
FSI: Economic Decline	-0.000	-0.055	0.211	-0.330
	(0.112)	(0.166)	(0.313)	(0.395)
FSI: Uneven Economic Development	-0.235	0.281	0.008	-0.132
	(0.187)	(0.353)	(0.370)	(0.383)
FSI: Human Flight and Brain Drain	0.454***	0.131	0.954**	-0.211
	(0.157)	(0.221)	(0.396)	(0.483)
FSI: State Legitimacy	0.100	0.675***	1.305***	1.187*
	(0.153)	(0.238)	(0.409)	(0.627)
FSI: Public Services	0.355**	0.047	0.003	0.776
	(0.156)	(0.174)	(0.409)	(0.653)
FSI: Human Rights and Rule of Law	-0.025	-0.587**	-1.109***	-0.234
	(0.138)	(0.281)	(0.371)	(0.720)
FSI: Demographic Pressures	0.390*	0.249	-0.021	-0.789
	(0.200)	(0.232)	(0.456)	(0.643)
FSI: Refugees and IDPs	-0.337*	-0.281*	0.031	-0.330
	(0.176)	(0.162)	(0.295)	(0.429)
FSI: External Intervention	-0.312*	0.010	0.658	0.816
	(0.163)	(0.176)	(0.528)	(0.554)
log(*Distance*^*o*,*d*^)	-1.305***	-0.692*		
	(0.237)	(0.413)		
$\text{log}(Diaspora_{t-12}^{o,d})$		0.414*		-2.974***
		(0.212)		(0.978)
Destination FE	Yes	Yes		
Origin-Destination FE			Yes	Yes
Month FE	Yes	Yes	Yes	Yes
Pseudo R^2^	0.57	0.49	0.74	0.69
N Obs.	2,726	1,152	2,679	1,152

Standard errors in parentheses, clustered by origin-destination pair

* *p* < 0.10,

** *p* < 0.05,

*** *p* < 0.01

#### The role of rescue probabilities

This analysis suggests that the probability of rescue has a large positive and significant relationship with attempted crossings: holding all else equal, a one percentage point increase in the probability of rescue is associated with a 6.7% increase in total crossings. If we use the more conservative coefficient in column (4), this still translates to a 5.5% increase in total crossings.

#### Other factors associated with crossings

As expected, we find that the number of attempted crossings is negatively correlated with the pairwise distance between the origin and destination countries. Furthermore, specification (2) suggests that there are generally a higher number of crossings between country pairs when there is already large diaspora community in the target country (although this coefficient reverses in sign when including country-pair fixed effects).

We also find a meaningful association between crossings and the Fragile States variables. There are more crossings from states that have higher levels of insecurity, violence, and crime (“security apparatus”) and threats to state legitimacy (“state legitimacy”). Interestingly, holding all else equal, there are fewer crossings on average from states with high levels of ethnic or other divisions (“factionalized elites”), repression (“human rights and rule of law”), and displaced populations (“refugees and IDPs”), although the latter relationship might result from the fact that the “refugees and IDPs” indicator also captures the extent to which a state hosts displaced populations (potentially, as a refugee receiving country).

#### Policy implications

In general, we interpret these findings with caution for several reasons. First, the FSI indicator variables are complex and highly correlated with each other (see Fig S2.1 of S2 Appendix in S1 File); for example, it is difficult to reason about the impact of factionalized elites independent of state legitimacy. Second, current values of the FSI indicators are also correlated with the size of a country’s diaspora, since the level of indicators may persist across years (e.g., states with uneven economic development in the current year likely also suffered from this problem in the previous year) and these indicators may drive people to migrate and form the diaspora. Third, there is uncertainty in the quantity of the dependent variable due to the fact that arrivals by land and sea are reported together and we do not have data to calculate separate rescue probabilities by country of origin. Nevertheless, taken together these results suggest a significant role of push factors in determining the number of migrant crossings, and they indicate that the number of crossings is correlated with rescue probability even after controlling for these factors.

While these results are illustrative, a final challenge with this model is that it is difficult to draw causal conclusions about the probability of rescue and the number of crossing attempts. In particular, the number of crossing attempts may be high because the probability of rescue is high, but rescue activity might also reflect the volume of attempted crossings, or both crossings and rescue activity could follow a common external trend. In the next section, we turn to an error correction model to estimate how crossings at the route level respond to rescue probability in the short and long run, in order to explore this causal relationship in more depth.

### Error correction model

Since the crossing trend is highly non-stationary, a naïve regression can misidentify the model due to the problem of spurious regressions [71]. To address this concern, we adopt a time-series error correction model to analyze the long- and short-term effects of rescue probability on the number of crossings [72]. Our results suggest that a reduced chance of successful crossing results in a smaller number of attempted crossings. We also analyze spillovers to the Western Mediterranean route, and find significant but limited substitution to this route.

#### Model

In this section we provide a brief overview of the ECM, following the exposition in [72, Section 6]. The development of the ECM was motivated by the observation that when running Ordinary Least Squares (OLS) regressions using non-stationary dependent and independent variables (in our case, *N*_*t*,*cross*_ and *P*_*t*,*rescue*_, respectively), there is an elevated risk of finding a significant relationship between the two even when none exists (i.e., a spurious regression [71]). One solution in this case is to take first differences in order to obtain stationary variables, and then to fit a regression on the first differences. However, this provides insights only about short-run relationships between the variables, and does not account for the fact that these variables may react to each other in the longer term. In fact, it is possible that two non-stationary series have a cointegrating relationship, in which a linear combination of the series is stationary (i.e. they have a stable long-run relationship). For example, it may be the case that in equilibrium:

$$\begin{array}{c} N_{t,cross}\;\;=\;\;\beta_{0}\;\;+\;\;\beta_{1}\;P_{t,rescue}, \end{array}$$ (1)

where *N*_*t*,*cross*_ is the monthly number of crossings (in thousands) and *P*_*t*,*rescue*_ is defined as above. The ECM essentially allows for both of these short- and long-run dynamics. Specifically, we estimate an ECM of the form:

$$\begin{array}{c} \Delta N_{t,cross}\;\;=\;\;\alpha_{0}\;\;+\;\;\alpha_{1}\;e_{t-1}\;\;+\;\;\alpha_{2}\;\Delta P_{t-1,rescue}\;\;+\;\;\epsilon_{t}, \end{array}$$ (2)

where Δ*N*_*t*,*cross*_ = *N*_*t*,*cross*_ − *N*_*t*−1,*cross*_; *α*_0_ is a constant term; *e*_*t*−1_ = *N*_*t*−1,*cross*_ − *β*_0_ − *β*_1_ *P*_*t*−1,*rescue*_, the observed deviations from equilibrium as defined by Eq 1; Δ*P*_*t*−1,*rescue*_ is defined analogously to Δ*N*_*t*,*cross*_; and *ϵ*_*t*_ is a random error term. In this case, *α*_1_ reflects the long run adjustment behavior that results from divergence between *P*_*t*,*rescue*_ and *N*_*t*,*cross*_, whereas *α*_2_ reflects the short run adjustment in *N*_*t*,*cross*_ that results from a change in *P*_*t*−1,*rescue*_.

We conduct our estimation using the Engle-Granger method [72, Section 6.3.1] in Stata SE 18.0; this is a two-step procedure in which Eq 1 is first estimated from the data and the lagged residuals are then used as an estimate for *e*_*t*−1_ in Eq 2.

### Results

#### Evidence that migrant crossings on the Central Route respond to changes in the probability of rescue

We begin by estimating the relationship between crossing behavior and the probability of rescue on the Central Mediterranean route. The maximum number of crossings ever observed on the route was 29,478 in October 2016. The results of estimating Eq 2 using the monthly differenced number of crossings (in thousands) as the dependent variable are shown in Table 2.

**Table 2:** Results of estimating the error correction model: Crossings on the Central route (in thousands) vs. rescues on the Central route.

	(1)
	$\Delta N_{t,cross}^{central}$
${\hat{e}}_{t-1}$	-0.402***
	(0.123)
$\Delta P_{t-1,rescue}^{central}$	-3.249
	(5.477)
Constant	0.060
	(0.698)
R^2^	0.200
R^2^—adjusted	0.163
N Obs.	46
Mean Dep. Var.	-0.07

Standard errors in parentheses

* *p* < 0.10,

** *p* < 0.05,

*** *p* < 0.01

We find significant evidence of long-run adjustment behavior, suggesting that the number of people crossing increases in the probability of rescue. When crossings and rescue probability diverge from their equilibrium relationship, adjustment occurs fairly quickly: our results suggest that the log number crossing falls by approximately 40% of the deviation from equilibrium in each period after the divergence. In other words, within four months over 85% of the adjustment needed to restore equilibrium has occurred. Interestingly, we find no significant evidence of a short-run relationship between crossings and rescue probability.

The equilibrium relationship is estimated to be:

$$\begin{array}{ccc} N_{t,cross}^{central} & \;=\; & -10.58\;\;+\;\;28.01\;P_{t,rescue}^{central}. \end{array}$$

To place this equation in context, during our period of observation, we have seen the probability of rescue fall from approximately 90% to 50%. In the long run, this is expected to correspond to a reduction of about 11,200 people per month according to our model (that is, (28.01 × 0.9) − (28.01 × 0.5)).

**Robustness checks**: We conducted Dickey-Fuller tests and Engle-Granger cointegration tests to verify the appropriateness of the error correction model, which we discuss in S2 Appendix in S1 File. In general, our results are robust to different choices of the dependent variable and to several different specifications of the model, which we present in S2 Appendix in S1 File.

#### Evidence that migrants substitute strategically from the Central to the Western Route

Next, we test whether the probability of rescue on the Central Mediterranean route affects crossings on the Western Mediterranean route through Spain. As above, we gather flow data from IOM, which reports the monthly number of sea arrivals in Spain as well as the estimated number of deaths along the Western Mediterranean route. Note that, as discussed above, interception data for coast guards along the Western Mediterranean route is not included in the IOM dataset (presumably because it is not available to IOM) so the number crossing includes only those who are rescued and those who are reported dead or missing. The maximum number of monthly crossings for the Western Mediterranean route was 10,598 in October 2018.

In Table 3, we re-estimate the error correction model in Eq 2 using the differenced number of crossings (in thousands) on the Western route as the dependent variable. As above, our estimates suggest significant long-run adjustments in response to deviation from the equilibrium relationship between Western Mediterranean crossings and the Central Mediterranean probability of rescue. However, the speed of adjustment is considerably slower (20% vs. 40%). As before, we find no significant short-term effect of the probability of rescue on crossings.

**Table 3:** Results of estimating the error correction model: Crossings on the Western route (in thousands) vs. rescues on the Central route.

	(1)
	$\Delta N_{t,cross}^{western}$
${\hat{e}}_{t-1}$	-0.204**
	(0.092)
$\Delta P_{t-1,rescue}^{central}$	0.421
	(1.525)
Constant	0.040
	(0.199)
R^2^	0.102
R^2^—adjusted	0.060
N Obs.	46
Mean Dep. Var.	0.03

Standard errors in parentheses

* *p* < 0.10,

** *p* < 0.05,

*** *p* < 0.01

The equilibrium relationship is estimated to be:

$$\begin{array}{ccc} N_{t,cross}^{western} & \;=\; & 5.32\;\;-\;\;4.31\;P_{t,rescue}^{central} \end{array}$$

This suggests a negative relationship between the two: as the probability of rescue along the Central Mediterranean route decreases, Western Mediterranean crossings increase. In particular, if the probability of rescue falls from approximately 90% to 50%, this model predicts about 1,700 additional crossings per month (that is, (4.31 × 0.9) − (4.31 × 0.5)). Therefore, in addition to the slower rate of adjustment, we find that the number of crossings on the Western Mediterranean route exhibits a smaller absolute response to changes in the probability of rescue on the Central route. Taken together, our models estimate that when the probability of rescue on the Central Mediterranean route falls from 90% to 50% (roughly corresponding to the shift between mid 2016 and mid 2019), approximately 15% of the 10,300 people who are deterred from crossing on the Central Mediterranean route will shift to the Western Mediterranean route. As noted above, this may be an underestimate because our estimate of Western Mediterranean crossings does not include data on interceptions.

## Analysis of the individual incident dataset

### Materials and methods

Thus far, we have analyzed the data on migration flows over the Central Mediterranean route and found that migrants’ decisions to cross on the Central Mediterranean route exhibit a long-term response to changes in the probability of rescue.

While migrants make a strategic decision of whether and where to cross depending on crossing conditions, smugglers may also respond to these conditions by varying their strategy. As the LCG claimed increasing control over the coastal zone, migrant boats located in the Libyan SAR zone were more likely to be intercepted and less likely to be rescued to Europe. Consequently, we observe two high-level shifts in the strategic actions of smugglers along the Central Mediterranean route.

First, the use of wooden boats increased, and the average size of boats departing from Libya (as measured by the number of people on board) grew smaller in this phase. The more aggressive the LCG’s interception activities are, the greater the distance migrant boats need to cross to secure a rescue by NGOs or European authorities. As a result, anecdotal reports suggest that smugglers have been using space on boats to load more fuel [36], rather than collecting more profit (and potentially, slowing boats down) by adding additional passengers.

Second, the share of boats departing from Tunisia relative to Libya, which was very small at the beginning of Phase 3, increased. In this section, we focus on boats departing from Libya because it is not clear whether local smuggling networks in Libya can choose to launch boats from Tunisia, and because the Libyan route has been historically more popular and has more total incidents than the Tunisian route.

We begin by describing the incident data below. Then, in the context of rubber boats, we build and estimate a choice model of Libyan smugglers’ strategic shift to smaller boats in response to LCG interceptions and the reduction in EU/NGO-led rescues. Using this model, we conduct counterfactual estimation of rubber boat sizes under different levels of Libyan interceptions, which explains the snowballing shift towards small boat sizes observed in 2018–2019. S3 Appendix in S1 File. provides further descriptive analysis of the incident data for context.

#### Description of the incident dataset

We use the term “incident” to refer to an attempted crossing of one or more migrants at sea, which has been entered as a record in a database. For our primary analysis, we collect incident data from Frontex, the European border control agency which supervises the deployment of aerial and naval assets to patrol the EU’s maritime borders. Frontex maintains the Joint Operations Reporting Application (JORA) database, which is a standardized information system for “incidents occurring during the entire cycle of joint operations coordinated by Frontex” [73]. These incidents are created with contributions from member state law enforcement with the goal of providing real-time situational insights [74].

In response to our request for public access to documents, Frontex has released records of incidents which occurred under Operations Hermes, Triton, and Themis from 2014–2019. Each incident corresponds to a boat or (occasionally) a set of multiple boats that is acknowledged by Frontex, and is reported with information including: the date of detection; the departure country of the migrants; the number of people involved; the number of deaths; the boat type; and whether the boat was detected inside or outside of Frontex’s operational area [75]. In total, the datasets we received contained 4,365 incidents originating in Libya from 2014–2019, including rescues involving actors outside of Frontex, such as NGOs and merchant ships. We excluded incidents where the transportation type was land-based (i.e. “bus”, “camper van”, “on foot”, and “car”). The dataset we used to estimate the choice model was further reduced to 1,851 incidents, because it was limited to: incidents occurring from 2016 onwards (since Libyan interception data is only available from 2016); incidents involving rubber boats; and incidents where the number of people on board and the number of vessels involved were reported (i.e., not missing).

The choice to focus on rubber boats was made for two primary reasons. First, rubber boats tend to have a more uniform physical size, which means that the number of people is a reasonable proxy for crowding; this is not the case for wooden boats, which may vary dramatically in their capacity. Second, while rubber boats can be imported cheaply, the supply of large wooden boats in the region has become increasingly scarce over time as these boats have sunk or been destroyed by anti-smuggling operations (see the section on “Smuggling operations and strategy” above); therefore, the choice of how many people to place on board a wooden boat may be exogenously affected by the scarcity of large ships. Restricting our analysis to rubber boats preserves the majority of incidents in the Frontex dataset: 74% of incidents originating in Libya involve rubber boats, relative to just 14% of incidents which involve wooden boats. Further details on the distribution of boat sizes and the relationship between boat size and quarterly interception probabilities are discussed in S4 Appendix in S1 File.

*Other datasets and analyses*. We have also gathered four other incident datasets (from Médecins sans Frontières, Watch The Med, Broadcast Warnings, and the IOM), which contain incidents recorded by a rescue NGO, monitoring efforts, emergency calls, and an international organization, respectively. After comparing these different datasets, we found that Frontex is the most comprehensive dataset in terms of volume. Therefore, we solely use the Frontex dataset for our main analyses and use the other datasets for supplementary analyses.

In S3 Appendix in S1 File, we conduct a brief comparative analysis of these different datasets which suggests that after 2017Q3, there was a decrease in boat size and an increase in the share of wooden boats compared with rubber boats. We also observe an increase in incident distance from the SAR border, which implies that migrants are traveling further before detection and that the mobility of the boats is increasingly important. Finally, we confirm the relation between mobility and boat size, showing that the probability of boats being found in the Frontex operational area (i.e., farther from the coast of Libya) is higher when the boat size is small.

#### Strategy model for the shift in boat size

A key challenge with this incident dataset is that it is not a representative sample of all movements in the region. In particular, as noted above, the Frontex data does not cover LCG interceptions. (Note that some of the less comprehensive datasets mentioned above may include individual LCG interception incidents; see S3 Appendix in S1 File for details.) As a result, we do not have the data to directly estimate how a given set of inputs (e.g. boat type or boat size) translates to outcomes (i.e. rescue, interception, or going missing/death) at the incident level.

Instead, we proceed in two stages. First, we identify the overall probability of interception in a given quarter. Then, we estimate how the behavior of smugglers changes as a function of the quarterly interception probability. The smuggler can influence his or her chance of interception for a given incident by altering the size of boat, trading off between the profits from adding more passengers and a reputational risk of interception that is expected to rise when boats are overcrowded and the prevailing rate of interception is high. By observing the actual choices of smugglers, we attempt to infer the parameters of their utility function.

Formally, our model of the smuggler’s utility depends on two primary factors: the number of people on board a given boat, and the estimated probability that a boat will be intercepted in the Libyan SAR zone. We aggregate the interception data by quarter because this secures a sufficient number of incidents observed per quarter, and because we estimate that three to four months is approximately the amount of time it takes for crossing behavior to react to changes in the probability of rescue (see the section entitled “Analysis of the aggregate flow dataset” above for further discussion on the rate of adjustment), which suggests that this may also be a sensible time scale at which to analyze smuggler responses.

*Estimating the probability of interception in the Libyan SAR zone*. We begin by estimating the quarterly probability that a boat departing from Libya is intercepted, $p_{intercept}^{qL}$, where *q* denotes the quarter and *L* denotes the fact that the boat originated in Libya. This part utilizes the flow data as well as the incident data. While the IOM flow data includes the number of interceptions off the coast of Libya $\left(N_{intercept}^{qL}\right)$, it includes only the *total* number of arrivals ($N_{rescue}^{q})$ and deaths $\left(N_{death}^{q}\right)$ for the Central Mediterranean route, which might include incidents originating in nearby countries such as Egypt or Tunisia. To proceed with the estimation of $p_{intercept}^{qL}$, we therefore rely on the incident-level data from Frontex and make the following two assumptions:

1. *Frontex’s recorded incidents do not cover interceptions. However, Frontex data is a representative and nearly comprehensive sample of rescue incidents in the region*. This assumption is justified by the fact that the total population in the Frontex dataset generally matches the total number of arrivals reported by IOM (see Fig S4.1, S4 Appendix in S1 File).
2. *The smuggler’s decision focuses on the probability of interception and does not independently weigh the probability of sinking, which is small. Therefore, excluding this outcome will not substantially affect the model results*. This assumption is justified by the fact that the casualty rate is low relative to the number of interceptions and crossings (see Fig 3), and that migrants are typically very distressed by the prospect of LCG interception.

Additional discussion of these assumptions is provided in S4 Appendix in S1 File.

Using Assumption (1), the number of arrivals from Libya can be estimated as:

$$N_{rescue}^{qL}\;=\;s_{rescue}^{qL}\;*\;N_{rescue}^{q},$$

where $s_{rescue}^{qL}$ is the quarterly share of migrants rescued who departed from Libya as opposed to other departure countries, which we estimate from the Frontex data. Using assumption (2), the smuggler’s expected probability of interception given departure from Libya is:

$$p_{intercept}^{qL}\;=\;\frac{N_{intercept}^{qL}}{N_{rescue}^{qL}\;+\;N_{intercept}^{qL}}.$$

The calculated probability of interception over time is illustrated in Fig 7. For all remaining sections of the paper, we simplify the notation by removing the *L* superscript and use $p_{intercept}^{q}$ to refer to the estimated rate of interception for boats departing Libya only.

**Fig 7:**
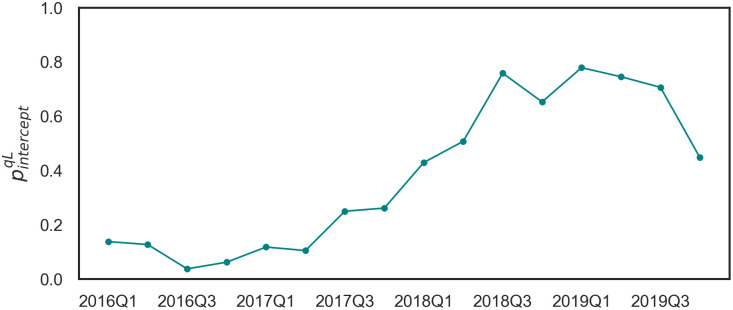
The estimated probability of interception for boats departing Libya by quarter.

*The smuggler’s utility function*. Having calculated the quarterly interception probability, we proceed to analyze incident-level decision-making. As noted above, we restrict our analysis to incidents involving rubber boats, although a similar utility function (with different coefficients) could be estimated using data on wooden boats. We next assume that the smuggler makes a choice of boat size. We discretize boat size into bins of *n* ∈ {1 − 50, 50 − 100, 100+} migrants. The smuggler then selects *n* to maximize his payoff according to his utility function, which takes the form:

$$\begin{array}{c} U_{in}\;=\;\alpha_{n}\;+\;\beta_{n}\;p_{intercept}^{q}\;+\;\varepsilon_{in}. \end{array}$$ (3)

Here, *i* represents the choice setting; *n* represents the choice of boat size; *α*_*n*_ represents a boat size fixed effect; $p_{intercept}^{q}$ is the estimated quarterly probability that a boat departing Libya will be intercepted in the Libyan SAR zone, for the quarter that corresponds to incident *i*; *β*_*n*_ is a boat size-specific coefficient on the quarterly probability of interception; and *ε*_*in*_ is a choice-specific idiosyncratic error term.

In other words, the smuggler’s utility can be interpreted as a function of two deterministic factors:

1. **The short-run monetary payoff** to the number of migrants chosen (*α*_*n*_), i.e. the smuggler’s valuation of the profit collected from launching a boat with *n* migrants on board. We generally expect this profit to increase in *n*, since each passenger is charged a fare for the journey. Evidence suggests that the fees for Central Mediterranean sea crossings are typically paid in advance—sometimes via extortion of migrants’ families—so we assume that this payoff is collected regardless of whether or not a journey is successful [3, 76, 77]. We also make the simplifying assumption that both the fee paid by migrants and the cost of a standard rubber raft is fixed over time; while this is unlikely, it is difficult to construct a more nuanced estimate of smugglers’ profit structures given the variability of prices reported in the literature (see Section 4.5 of S4 Appendix in S1 File for further discussion).
2. **The expected long-run reputational payoff** of crossing ($\beta_{n}\;p_{intercept}^{q}$), which depends on the smugglers’ risk of interception. Since the probability of interception should vary by boat size, but the precise extent of variation is unknown, we allow for boat-size-specific coefficients on this probability. These coefficients are effectively an estimate of how changes in the interception probability affect utility for different choices of boat size. If small boats provide an advantage when interceptions are high, we expect *β*_0−50_ > *β*_50−100_ > *β*_100+_, whereas if large boats provide an advantage we expect the opposite to be true. We assume that reputation has the potential to yield profits for the smuggler in the long term, because evidence suggests that trust plays an important role in migrants’ choice of smugglers, and that migrants research smugglers before engaging them—often relying on word-of-mouth, personal recommendations, or social media, including testimonials of those who have crossed successfully [76, 78, 79].

The addition of an idiosyncratic error term *ε*_*in*_ allows for random variation in the behavior of smugglers due to unobservables, such that not all smugglers will select the same boat size choice even under the same conditions. For example, this term could include unobserved variation in smuggler costs, resources, or preferences. While in theory a single smuggler could be associated with multiple incidents, we do not have information about how incidents are associated with individual smugglers (or even the extent to which smuggling efforts are coordinated [77]) and so each incident is assumed to be associated with a different smuggler and incidents are assumed to be independent of each other. Due to the inclusion of this error term *ε*_*in*_, this model is a type of random utility maximization model.

Assuming that *ε*_*in*_ takes a Type-I extreme value distribution, the probability of choosing boat size *n* takes the form of the standard logit probabilities [80, Section 3.1]. Let N={1-50,50-100,100+} be the set of possible boat size choices and let *V*_*in*_ = *U*_*in*_ − *ε*_*in*_; that is, let *V*_*in*_ represent the deterministic part of the utility function. Then,

$$\begin{array}{c} P_{in}\;=\;\frac{e^{V_{in}}}{\sum_{n^{\prime }\in N}\;e^{V_{in^{\prime }}}}. \end{array}$$ (4)

This model is also sometimes referred to as the Maximum Entropy Inverse Reinforcement Learning (IRL) model [59–61], in which the smuggler’s choice of boat size is drawn proportional to his exponentiated expected reward:

$$\begin{array}{c} P_{in}\left(\alpha_{n},\beta_{n}\right)\;\propto \;e^{\;V_{in}\left(\alpha_{n},\beta_{n}\right)}. \end{array}$$

#### Estimation of the utility function from incident-level data

Using the model described in Eqs 3 and 4, we empirically estimate the parameters *α*_*n*_ and *β*_*n*_ in Eq 3. Recall that *α*_*n*_ describes the payoff to a given boat size which is independent of $p_{intercept}^{q}$, whereas *β*_*n*_ describes the long-term payoff associated with the probability of interception vs. rescue, conditional on boat size. We attempt to recover *α*_*n*_ and *β*_*n*_ using incident-level data from Frontex.

In our model, the log likelihood of the data is:

$$\begin{array}{c} \text{log}\;L\left(\alpha ,\beta \right)\;=\;\sum_{i\in I}\;\sum_{n\in N}\;\;d_{in}\;\;\text{log}\left(\;P_{in}\left(\alpha_{n},\beta_{n}\right)\;\right), \end{array}$$ (5)

where I is the set of incidents in the dataset and *d*_*in*_ is equal to one if *n* is the boat size actually chosen in incident *i*, and zero otherwise. Estimation was performed in Stata SE 18.0 using the clogit command, which optimizes the log likelihood function using Newton’s method.

### Results

Results from the estimation of the model are shown in Table 4. Note that there are a limited number of observations in 2018 and 2019 compared with 2016 and 2017 (see Table S4.1 of S4 Appendix in S1 File for detailed information). For this reason, we re-weight all incidents such that each quarter in the dataset is given equal weight; for further discussion of the role of the weights and a comparison with unweighted estimates, see S4 Appendix in S1 File. Because choice probabilities are relative, we must fix the utility of one choice category in order to identify the others. We normalize the utility of the 0−50 boat size category by setting *α*_0−50_ and *β*_0−50_ to zero. The remaining coefficients are then interpreted in relation to this base category.

**Table 4:** Parameter estimates for the smuggler’s utility model.

	*p*_*in*_
*α*_50−100_	1.786***
	(0.413)
*α*_100+_	3.849***
	(0.604)
*β*_50−100_	-3.587***
	(0.955)
*β*_100+_	-6.511***
	(1.998)
pseudo *R*^2^	0.268
N choice alternatives	5,553
N choices	1,851

Standard errors in parentheses

* *p* < 0.10,

** *p* < 0.05,

*** *p* < 0.01

We can see that when there is no chance of interception, larger numbers of passengers per boat are generally preferred (*α*_0−50_ < *α*_50−100_ < *α*_100+_). This is consistent with our expectations, since smugglers can likely extract a higher rent by launching more crowded rubber boats. However, our results suggest that boats with larger numbers of passengers face a growing disadvantage when the probability of interception is nonzero (*β*_0−50_ > *β*_50−100_ > *β*_100+_). This is consistent with the empirical evidence that large boats are chosen less frequently when interceptions are high, as they may struggle to evade the LCG and reach the EU SAR zones. Taken together, these results support the hypothesis that there is a tradeoff between the short-run payoff to launching large boats and the long-run cost to interception as boats grow more crowded.

In Fig 8 we compare our model-based estimates for the distribution of boat size to the empirical distribution of boat sizes by quarter. We can see that generally, the predictions of the model follow the overall trends in the empirical distribution of boat sizes. Although we do not fully match the fluctuations in average boat size by quarter that occur from 2018 onward, we note that these fluctuations occur in part because of the small number of incidents in the last two years of our dataset.

**Fig 8:**
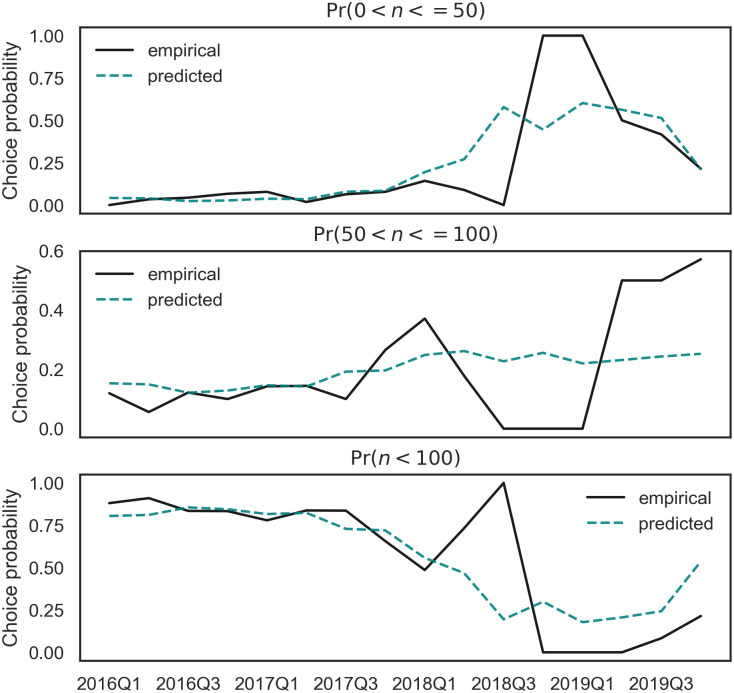
Predicted distribution of boat sizes by quarter.

#### Robustness checks

We find that the drop in the number of passengers per boat between Phase 2 and Phase 3 is statistically significant, which we detail in S4 Appendix in S1 File. We also consider different weighting schemes in S4 Appendix in S1 File.

#### Counterfactual estimation

Equipped with these model estimates, we can conduct simulations to illustrate how smugglers are expected to react to a change in interception rates. Using the incident-level parameter estimates for *α*_*n*_ and *β*_*n*_, Fig 9 shows how the expected utility (i.e., the deterministic component of the utility) and choice probability for each boat size varies with the quarterly rate of interceptions. We estimate that large boat sizes are preferred until the interception rate approaches 60%, which occurs starting in the third quarter of 2018 (see Fig 7). After this point, small boats are preferred. Interestingly, midsize boats are never the dominant choice in expectation.

**Fig 9:**
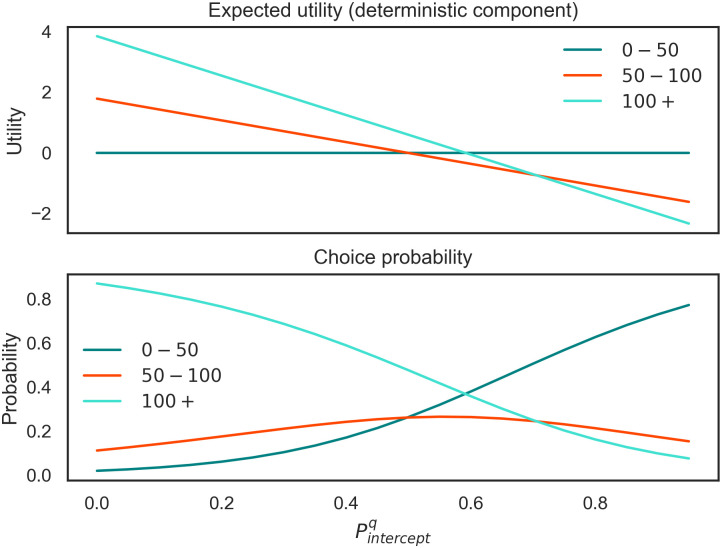
Predicted utility and distribution of boat sizes, by probability of interception.

Fig 10 illustrates a hypothetical scenario for rubber boats in which the number of incidents per quarter remains the same, but the probability of interception is increased by 10 percentage points (relative to the baseline interception rate) across all quarters. Using the model and these two different interception levels, we estimate the choice probabilities for each scenario. Across all quarters in our dataset, we predict that the smugglers will respond to the changing environment by increasing the use of small boats and decreasing the use of large boats; the effect on the use of mid-size boats varies by period.

**Fig 10:**
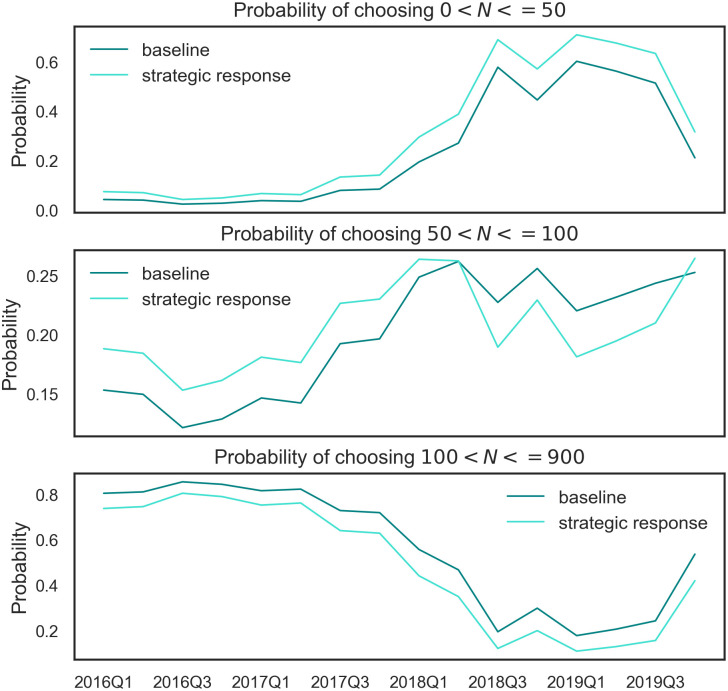
Predicted distribution of boat sizes, given a 10 percentage point increase in the probability of interception. “Baseline” refers to the “true” scenario in which the interception rate matches reality. “Strategic response” refers to the scenario in which smugglers adapt their choice probabilities to reflect the higher probability of interception.

## Discussion and conclusions

Prior work on migration flows in the Central Mediterranean has focused on assessing the validity of the claims that NGO rescues endanger migrants by encouraging crossings and incentivizing riskier trips. In response to these claims, Italy and the EU more generally have acted to increase the capacity and responsibilities of the LCG and encourage a more aggressive regime of interceptions and returns. However, to date there has been little theoretical analysis of how migrants have responded to these changes in recent years.

In this paper, we analyze the flow of Central Mediterranean migrants over time, including their response to the rise in Libyan interceptions that started in mid-2017. From our ECM analysis of the flow data we find that the number of crossings on the Central route increases with the success probability of crossing, i.e., that the overall flow of migrants does respond to the growing rate of interceptions. Our analysis of the gravity model finds a similar relationship when modeling all three routes together, and suggests that this relationship is present even when controlling for push factors. Our ECM analysis also indicates spillovers from the Central Mediterranean route to the Western Mediterranean route, although we estimate that only a fraction of migrants substitute across routes and descriptive analysis suggests that the propensity to substitute appears to vary by geography of origin. These findings correspond to the deterrence and diversion effects previously identified in the literature [4].

Our analysis supports the claim that the growing rate of Libyan interceptions has discouraged migrant crossings on the Central Mediterranean route. However, crossings have continued despite a very high rate of interceptions (approaching almost 80% for boats departing Libya). Therefore, we also analyze how smugglers have adapted to the changing interception environment. When comparing incident-level datasets (S3 Appendix in S1 File), we observed evidence of strategic responses along several dimensions. Namely, there appears to be a decline in the number of people per boat and a shift towards the use of wooden boats. These strategy shifts coincide with an overall tendency for incidents to occur closer to the EU SAR zones, and we find a general correlation between boat type/boat size choice and incident locations in these later periods.

To formally analyze these changes, we build a utility model of smuggling using rubber boats. Using a random utility maximization model, we estimate that there is a positive payoff to launching larger/more crowded rubber boats, but that this is counterbalanced by a penalty on larger boats that rises with the overall rate of interceptions. Therefore smugglers trade off between collecting higher profits by continuing to crowd migrants on to boats, and the reputational costs of launching crowded boats that have a lower probability of success.

The trend towards the use of smaller boats may have several implications for migrants crossing on the Central Mediterranean route. On the one hand, boats with fewer passengers may be less likely to sink, since overcrowding can make boats less seaworthy. For example, overcrowded boats sit low in the water and may be more likely to take on waves. In rubber rafts, overcrowding risks the collapse of the inflatable tubes that support the boat, whereas wooden boats can tip if they are over capacity and passengers suddenly move to one side (e.g., in the course of a rescue). On the other hand, boats that are physically smaller in size may be less likely to be detected at sea and may be more susceptible to weather conditions on the open water. We do see a rise in the rate of deaths reported by the IOM along the Central Mediterranean route after 2017 (see Fig 3), suggesting that deaths may be occurring in the course of LCG interceptions and/or that ships are sinking without a rescue or interception ever being initiated.

Consistent with other findings in the literature, our work suggests that increased border enforcement induces strategic responses on a number of different dimensions, which can lead to unpredictable or unforeseen consequences for the crossing experience. When debating an increase in border surveillance, it is important to consider implications for both migrant and smuggler strategy, as well as the possible long-run impact of these strategy shifts on fatalities. In general, analyzing these implications is difficult because data on informal migration is typically incomplete and/or biased (if it is available at all), and because causal inference is challenging in such a complex environment. We aim to address the latter challenge through the use of an error correction model to reduce the risk of spurious regressions in the flow data, and address the former by combining biased incident data with more representative flow data in our random utility maximization model. However, there is also the possibility of a strategic counter-response to smugglers on the part of the LCG, which we do not examine further in this context because of the lack of transparency on LCG activities.

Future work could expand along several different dimensions. First, there is a clear need for careful empirical work which collects and combines different sources of data in order to produce comprehensive migration incident datasets at the regional level. For example, we have experimented with the application of dataset matching methods from other fields, namely capture-recapture models [81], for this purpose. Second, existing strategy models could be expanded to more sophisticated settings, for example by building nested choice models in which smugglers choose a boat type before selecting a boat size, or select between departure ports before making boat-specific decisions.

While the Mediterranean is currently the world’s deadliest border, new maritime crossing routes have continued to emerge globally as vulnerable people flee economic and political hardship in Venezuela, escape ethnic persecution in Myanmar, and seek economic opportunity in the United States [82–84]. By integrating different methods and data sources we aim to contribute to a growing body of literature on sea crossings, providing evidence on how these crossings are similar to or different from land-based informal migration. We hope that a better understanding of crossing behavior and strategy can ultimately contribute to safer and more humane global border regimes.

## Supporting information

S1 File S1 Appendix. Legal protections for migrants recovered at sea. S2 Appendix. Supplementary details for the flow analysis. S3 Appendix. Analysis of additional incident datasets. S4 Appendix. Supplementary details for the incident-level analysis.
(PDF)
