# Supplementary material for: Strategic choices of migrants and smugglers in the Central Mediterranean sea
Source: PLoS One. 2024 Apr 19;19(4):e0300553. doi: 10.1371/journal.pone.0300553 (PMC11029657; doi:10.1371/journal.pone.0300553)
Supplement: S1 File — S1 Appendix. Legal protections for migrants recovered at sea. S2 Appendix. Supplementary details for the flow analysis. S3 Appendix. Analysis of additional incident datasets. S4 Appendix. Supplementary details for the incident-level analysis. (PDF) [file pone.0300553.s001.pdf]

Supporting information

S1 Legal protections for migrants recovered at sea

Table S1.1. International agreements governing the rescue of migrants and refugees at sea.

Agreement	Year	Provisions
The Convention Relating to the Status of Refugees, Ch. V, Art. 33 [1]	1951	"No Contracting State shall expel or return ('refouler') a refugee in any manner whatsoever to the frontiers of territories where his life or freedom would be threatened on account of his race, religion, nationality, membership of a particular social group or political opinion."
International Convention for the Safety of Life at Sea (SOLAS), Ch. V, Reg. 10* [2]	1974	"The master of a ship at sea, on receiving a signal from any source that a ship or air craft or survival craft thereof is in distress, is bound to proceed with all speed to the assistance of the persons in distress informing them if possible that he is doing so."
SOLAS Amendment [3]	2004	"This obligation to provide assistance applies regardless of the nationality or status of such persons or the circumstances in which they are found."
International Convention on Maritime Search and Rescue, Ch. 2 [4]	1979	"Parties shall ensure that assistance be provided to any person in distress at sea. They shall do so regardless of the nationality or status of such a person or the circumstances in which that person is found."
SAR Convention Amendment [3]	2004	"The Party responsible for the search and rescue region in which such assistance is rendered shall exercise primary responsibility for ensuring such coordination and cooperation occurs, so that survivors assisted are disembarked from the assisting ship and delivered to a place of safety, taking into account the particular circumstances of the case . . . "

* In other versions, this is Regulation 33.

S2 Supplementary details for the flow analysis

Below, we provide additional details on the estimation of the gravity and error correction models presented in the section on "Analysis of the aggregate flow dataset"in the main paper.

2.1 Supplementary details for the gravity models

In this section, we provide supplementary context on the gravity model. Table S2.1 lists the variables used in the gravity model along with their type and source. Fig S2.1 is a correlation plot of these variables, indicating that the FSI variables tend to be positively correlated with each other and the source country's distance from European destinations, and negatively correlated with the size of the existing pairwise diaspora in Europe. Because of these correlations, we recommend that the coefficients on the push factors in the gravity model be interpreted cautiously.

Table S2.2 conducts a robustness check by re-estimating the gravity model with OLS regression. In general, Poisson regression is the preferred estimation approach because it allows the dependent (flow) variable to take zero values and because coefficient estimates will be consistent even if the error term is heteroskedastic (which is not the case for the OLS estimates) [5, 6]. Encouragingly, the results from OLS estimation align well with the the results in the main paper, although the coefficients are slightly larger in magnitude than the ones from the main paper (note that the number of observations is the same across both tables, as we add 1 to any zero-valued flow in order to avoid dropping observations when taking the log).

2.2 ECM of crossings on the Central Mediterranean route

2.2.1 Checks on the Model

Dickey-Fuller tests are consistent with the hypothesis that the total crossings, the log total crossings, the log odds of crossing, and the probability of rescue are non-stationary but that their first differences are stationary, suggesting that the ECM is appropriate in this setting. Similarly, the Engle-Granger test supports the hypothesis of cointegrating relationships between (1) the total crossings and the probability of rescue, (2) the log

Table S2.1. Factors included in the gravity model

Factor type	Indicator	Source
Flow	Number of arrivals from source country to destination country	UNHCR [7]
Distance	Rescue probability	IOM [8]
	Geographic distance between countries	US ITC [9]
	Number of people in destination who were born in source country	EuroStat [10]
Push	Social cohesion - security apparatus	FSI [11]
	Social cohesion - factionalized elites	FSI [11]
	Social cohesion - group grievance	FSI [11]
	Economy - economic decline	FSI [11]
	Economy - uneven economic development	FSI [11]
	Economy - human flight and brain drain	FSI [11]
	Politics - state legitimacy	FSI [11]
	Politics - public services	FSI [11]
	Politics - human rights and rule of law	FSI [11]
	Society - demographic pressures	FSI [11]
	Society - refugees and IDPs	FSI [11]
	Society - external intervention	FSI [11]

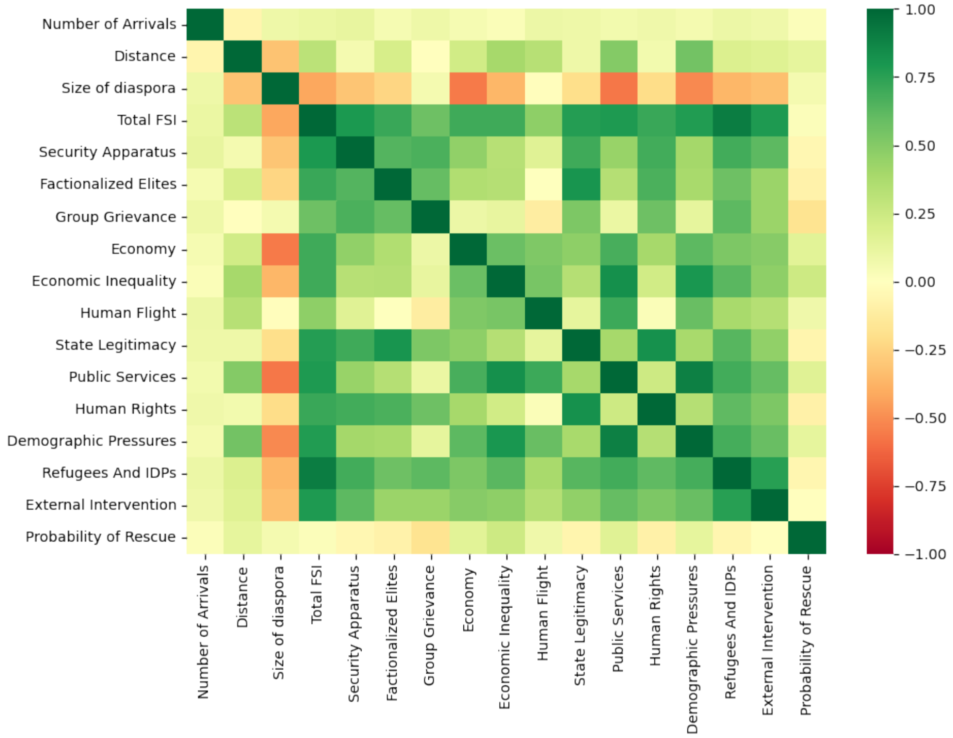

Fig S2.1. Correlation plot of the relationship between gravity model variables

total crossings and the probability of rescue, and (3) the log odds of crossing and the probability of rescue.

Table S2.2. Results of estimating the gravity model with OLS rather than PPML

	$\log(N^{o,d}_{cross,t})$			
	(1)	(2)	(3)	(4)
$P^{route}_{rescue,t-1}$	7.589***	9.272***	7.482***	8.956***
	(0.950)	(1.711)	(0.937)	(1.088)
FSI: Security Apparatus	0.067	0.538***	-0.693	-0.942
	(0.154)	(0.182)	(0.433)	(0.672)
FSI: Factionalized Elites	0.018	-0.178	0.722	1.396
	(0.182)	(0.168)	(0.698)	(1.365)
FSI: Group Grievance	0.206	-0.450*	-0.638	-0.731
	(0.167)	(0.227)	(0.456)	(0.874)
FSI: Economic Decline	-0.170	-0.126	-0.199	-1.035
	(0.167)	(0.260)	(0.486)	(0.784)
FSI: Uneven Economic Development	-0.424	0.272	0.060	-0.260
	(0.268)	(0.445)	(0.477)	(0.603)
FSI: Human Flight and Brain Drain	0.491***	0.251	0.526	0.012
	(0.172)	(0.288)	(0.434)	(0.901)
FSI: State Legitimacy	0.151	1.032***	0.662	0.503
	(0.253)	(0.305)	(0.667)	(0.906)
FSI: Public Services	0.179	-0.548*	0.286	0.313
	(0.276)	(0.281)	(0.427)	(0.955)
FSI: Human Rights and Rule of Law	-0.225	-1.244***	-0.618	0.717
	(0.272)	(0.346)	(0.654)	(1.077)
FSI: Demographic Pressures	0.276	0.438	-0.168	-0.540
	(0.278)	(0.341)	(0.520)	(1.095)
FSI: Refugees and IDPs	-0.119	0.232	0.317	0.011
	(0.262)	(0.214)	(0.425)	(0.701)
FSI: External Intervention	-0.034	0.069	1.438**	1.574
	(0.190)	(0.199)	(0.699)	(1.304)
$\log(Distance^{o,d})$	-1.725***	-0.969*		
	(0.412)	(0.524)		
$\log(Diaspora^{o,d}_{t-12})$		0.588***		-1.388
		(0.205)		(1.123)
Destination FE	Yes	Yes		
Origin-Destination FE			Yes	Yes
Month FE	Yes	Yes	Yes	Yes
Adjusted R^2	0.44	0.41	0.67	0.57
N Obs.	2,726	1,152	2,726	1,152

Standard errors in parentheses, clustered by origin-destination pair
* $p < 0.10$, ** $p < 0.05$, *** $p < 0.01$

2.2.2 Alternative specifications for the ECM of crossings on the Central Mediterranean route

Below, we discuss possible alternative specifications for the model presented in Eqs 1 and 2 of the main paper. In Table S2.3 of this appendix, we test three different measures of crossing behavior as the dependent variable: the differenced total number of

crossings in thousands, which is the dependent variable used in the main paper (columns 1-3); the differenced log number of crossings in thousands (columns 4-6); and the differenced log odds of crossing (columns 7-9).[1] We also experiment with different specifications of the ECM; the model described in Eq 2 is shown in columns 2, 5, and 8, but we also present the results when excluding the short-run adjustment term (columns 1, 4, and 7) and including lagged crossing behavior (columns 3, 6, and 9).

We estimate a similar speed of adjustment across all dependent variables: in each period after a divergence from the equilibrium relationship between crossing behavior and the probability of rescue, we estimate that the number of crossings falls by approximately 40% of the deviation from equilibrium, whereas the log number of crossings falls by approximately 43% of the deviation from equilibrium and the log odds of crossing falls by approximately 46% of the deviation from equilibrium. The estimated speed of adjustment is slightly slower when using a model that omits the differenced probability of rescue from the previous period (the short-run effect), and slightly faster when we also include the differenced dependent variable from the previous period. Our coefficients on the long-run speed of adjustment parameter remain significant across all specifications, and we never estimate a significant short-run adjustment.

The estimated equilibrium relationships for the log number of crossings and the log odds of crossing are:

$$\log\left(N_{t,cross}\right) = -1.17 + 4.13 \; P_{t,rescue} \quad (1)$$

$$\log\left(\frac{P_{t,cross}}{P_{t,stay}}\right) = -5.33 + 5.70 \; P_{t,rescue}. \quad (2)$$

These equations suggest that when the probability of rescue falls from approximately 90% to 50%, the number of monthly crossings will decline by approximately 10,300 - 12,200 people.

[1] In order to calculate the odds of crossing relative to staying, it is necessary to know how many people could potentially cross in a given period. We assume that the maximum potential number of people crossing is equal to the largest number of crossings observed in any period (29,478 for the Central Mediterranean route), multiplied by $\frac{10}{9}$ to ensure that there is no period in which all potential crossings occur (because this would result in division by zero when calculating the odds).

Table S2.3. Alternative specifications of the error correction model for crossings along the Central Mediterranean route.

	$\Delta \mathrm{N}^{central}_{t,cross}$			$\Delta \log(\mathrm{N}^{central}_{t,cross})$			$\Delta \mathrm{log\_odds}^{central}_{t}$		
	(1)	(2)	(3)	(4)	(5)	(6)	(7)	(8)	(9)
$\hat{e}_{t-1}$	-0.351***	-0.402***	-0.489***	-0.365***	-0.426***	-0.432***	-0.394***	-0.462***	-0.513***
	(0.115)	(0.123)	(0.137)	(0.120)	(0.133)	(0.150)	(0.122)	(0.133)	(0.152)
$\Delta \mathrm{P}^{central}_{t-1,rescue}$		-3.249	-4.206		-0.340	-0.356		-0.679	-0.847
		(5.477)	(5.464)		(0.650)	(0.683)		(0.982)	(1.015)
$\Delta \mathrm{N}^{central}_{t-1,cross}$			0.210						
			(0.152)						
$\Delta \log(\mathrm{N}^{central}_{t-1,cross})$						0.013			
						(0.159)			
$\Delta \mathrm{log\_odds}^{central}_{t-1}$									0.111
									(0.156)
Constant	-0.040	0.060	0.098	-0.024	-0.009	-0.009	-0.023	-0.002	0.002
	(0.685)	(0.698)	(0.691)	(0.078)	(0.079)	(0.080)	(0.120)	(0.122)	(0.123)
R^2	0.172	0.200	0.235	0.171	0.202	0.202	0.189	0.222	0.231
R^2 - adjusted	0.153	0.163	0.180	0.153	0.164	0.145	0.170	0.186	0.177
N Obs.	47	46	46	47	46	46	47	46	46
Mean Dep. Var.	-0.10	-0.07	-0.07	-0.04	-0.03	-0.03	-0.04	-0.03	-0.03

Standard errors in parentheses
* $p < 0.10$, ** $p < 0.05$, *** $p < 0.01$

2.2.3 Stability of coefficient estimates and backtesting

In addition to testing alternative specifications of the model, we also tested the robustness of the model to being fit on different time windows, in order to gain insight into the stability of coefficient estimates.

In Fig S2.2a of this appendix, we re-estimate the speed of adjustment over different expanding windows, starting with the five-month window from January - May 2016 and ending with the full 48-month dataset spanning January 2016 - December 2019. This allows us to determine how the coefficient estimate changes as more (recent) data is added to the model. As we can see, the estimated speed of adjustment has decreased[2] over time but appears to be stabilizing as more data points are used for estimation, suggesting that we are approaching a more consistent estimate.

The stability of coefficient estimates is particularly important for cases in which such estimates could be used to predict future behavior. Therefore, in Fig S2.2b of this appendix, we compare the observed and predicted period-to-period changes in the number of crossings along the Central Mediterranean route. We train the model on the years 2016-2018 and then predict the period-to-period changes in crossings in 2019 using the trained ECM model. For each data point, we then take the true observed arrivals

[2] Recall that the absolute magnitude of the rate of adjustment determines the speed whereas the sign determines the direction of the adjustment, so a less negative rate of adjustment is a "slower" rate of adjustment.

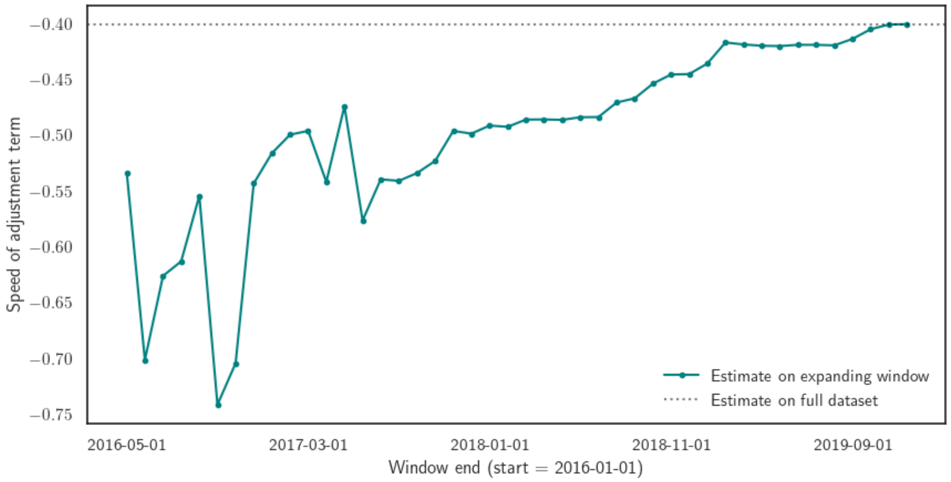

(a) Rate of adjustment, estimated over different expanding windows

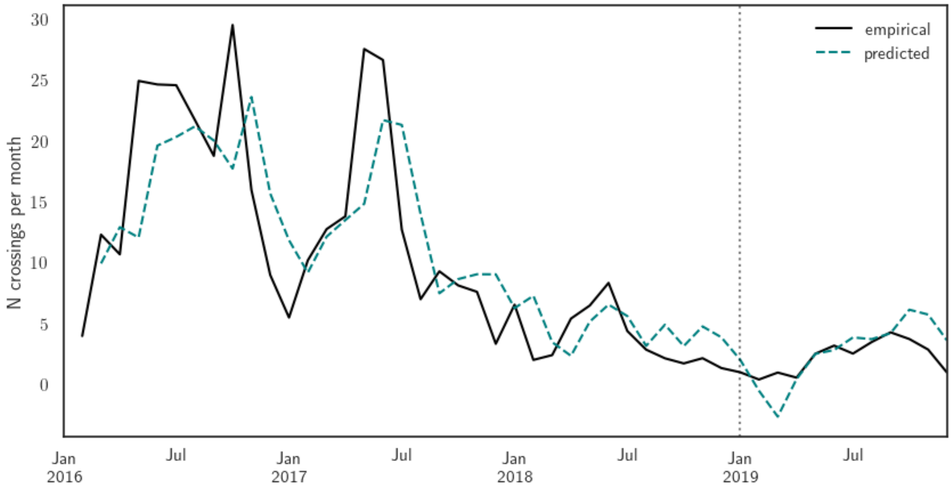

(b) Observed vs. predicted absolute number of crossings

Fig S2.2. Backtesting for the ECM model of crossings on the Central Mediterranean Route.

from period $t-1$ and add $\Delta\hat{N}^{central}_{t,cross}$ as predicted by the model to determine the estimated number of arrivals. As shown in the figure, we are able to approximate the observed arrivals trend; the mean absolute error is 1,949 arrivals on the training dataset (relative to a monthly average of 11,226 arrivals) and 1,141 arrivals on the test dataset (relative to a monthly average of 2,199 arrivals). While the model is fitting the training dataset, predictive power appears limited as the MAE from this ECM model is higher than that from the naïve approach of predicting no change since the previous period.

2.3 Substitution from the Central to the Western route

2.3.1 Checks on the model

Dickey-Fuller tests suggest that the number of crossings (in thousands) in the Western Mediterranean is also non-stationary, and that its first differences are stationary. However, we do not find significant evidence of a cointegrating relationship according to the Engle-Granger test; given that we have only 48 observations for training the model, we may be under-powered to detect such a relationship. We also note that this model seems less stable; [12] suggest that when the independent and dependent variables in the equilibrium equation are reversed and the ECM is re-estimated, the long-run adjustment term should remain significant, which is not the case here. While we fit an ECM for comparability with the model of crossings on the Central Mediterranean route, the results of this model in the Western Mediterranean context should be interpreted cautiously.

2.4 Substitution from the Central route to the Eastern route

When estimating the ECMs, we chose not to model the substitution from the Central route to the Eastern route for the following reasons.

First, our focus is on the Central Mediterranean route, which is primarily used by African migrants. When examining the flows data for African countries during our period of interest (Figs 4 to 6 of the main paper), we found very little evidence of substitution to the Eastern route by African migrants – only Algerians appear to have alternated over all three routes at different points in time. Conversely, when examining Asian and Middle Eastern source countries (Fig S2.3), we see that the three main source countries (Syria, Afghanistan, and Iraq) heavily favored the Eastern route, with little substitution to the Western/Central route. Only Pakistanis appeared to alternate between the Greek and Italian route.

Second, we believe that switching from the Western/Central route to the Eastern route is less frequent because of visa/travel restrictions. If we examine the main northbound migrant pathways (see for example, https://www.economist.com/sites/default/files/images/2016/10/blogs/graphic-detail/20161022_wom928.png or https://www.rhipto.org/_files/ugd/655326_b89cad5c8d4a4716ae0212f276029c26.pdf),

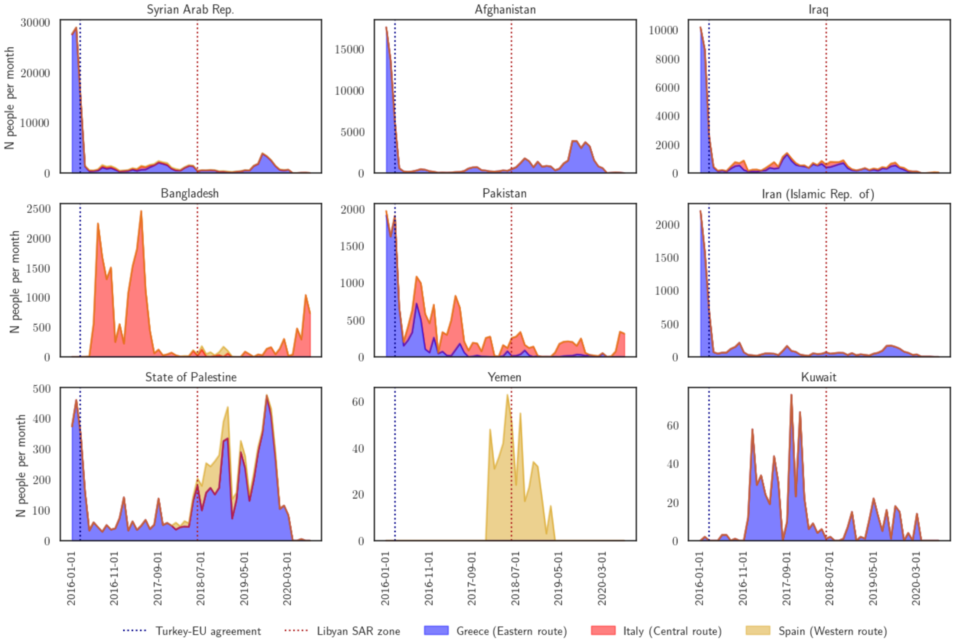

Fig S2.3. The number of migrants crossing by nationality and route: Asia and the Middle East. Data source: [63].

we see that there is relatively good connectivity from West Africa to the Western/Central Mediterranean departure points, whereas pathways to the Eastern route are fewer and less direct. As discussed in the main paper, mobility across West Africa is facilitated by freedom of cross-border movement under ECOWAS.

Finally, traffic to the Eastern route declined sharply from its 2015 peak after an agreement was reached to return irregular migrants from Greece to Turkey if they were not granted asylum [13]. For these reasons, we believe that the Eastern route became a less attractive alternative for migrants during our period of interest.

S3 Analysis of additional incident datasets

3.1 Supplementary data sources

As described in the section on "Analysis of the individual incident dataset" in the main paper, our primary analysis relies on incident-level data from Frontex. In these appendices, we draw on additional datasets to conduct supplementary analysis. Specifically, we gather further data on incidents from four primary data sources:

- **Watch The Med/Alarm Phone:** Watch The Med (WTM) is a platform that has monitored migration incidents in the Mediterranean since 2012 [14]. Watch The Med data comes primarily from Alarm Phone, an emergency telephone hotline designed to help migrants and refugees at sea. Alarm Phone typically receives calls directly from migrant boats that have been equipped with a satellite phone, which also allows these boats to report their positions. Incident reports may contain information on the ship location and type, the number of people on board, the port of departure, and the details of the rescue.

- **Broadcast Warnings:** Broadcast Warnings are a form of general-purpose maritime communication, and are issued to alert ships of nearby operations, dangers, and emergencies. They contain announcements about ships in distress, often reporting the location, the number of people on board, and a description of the situation. We parse the text of all Broadcast Warnings issued for the Mediterranean since 2014, removing irrelevant incidents [15].[3]

- **Médecins sans Frontières:** Data on rescues involving the NGO Médecins sans Frontières (MSF, or Doctors without Borders) is available through its online search and rescue portal, which tracks the activity of the organization's eight rescue ships since 2015 [16]. This portal contains information on rescue missions and individual operations, including the location and time of the rescue; the number of people involved; and the boat type and weather conditions.

- **Missing Migrants Project (IOM):** Finally, we incorporate data from the

[3]For example, we removed incidents that referred to hazards or logistics (e.g., cable-laying operations or adrift fishing gear) and ship-related incidents that did not appear to be related to migration (e.g., disabled fishing vessels or ships that were referenced by name). Incidents were first filtered heuristically using keyword searches, and remaining incidents were then manually inspected for relevance.

IOM's Missing Migrants Project, an initiative which has tracked migration-related deaths since 2014 [17]. The project collects data from national authorities, NGOs, and media sources to identify individual incidents. In particular, the missing migrants dataset contains information on the location of the incident, the number of dead or missing, the number of survivors, and the estimated cause of death.

Where geospatial information on incident locations is available, we restrict our dataset to incidents which occurred within the Italian, Maltese, or Libyan rescue zones.

Table S3.1. Overview of the incident-level datasets.

Data source	Collected by	Time span	N	Description	Limitations
Frontex	Frontex	2014-01 – 2019-12	4,365	Boat detections and interceptions recorded by Frontex, including incidents coordinated by national authorities, NGOs, and commercial ships.	This seems to be the most comprehensive incident-level dataset. However, many incidents are missing precise location data, and the dataset does not appear to include Libyan interceptions. Sometimes, multiple boats are reported as part of the same incident.
Watch The Med	Alarm Phone (primarily)	2014-06 – 2019-12	325	Incident reports that are largely drawn from calls for help to the Alarm Phone NGO hotline. Alarm Phone has no rescue capacity but it advocates on behalf of migrants, tracks their progress, alerts rescue authorities, requests intervention, and tops up the credit of boats' satellite phones so they can continue making calls.	Calls typically come only from boats equipped with satellite phones, or from relatives on shore. Calls to Alarm Phone seem to be *lower* in phases of high rescue activity, possibly because (1) many boats were independently detected by rescue NGOs in the region without a call; and (2) migrants may have been more likely to call the MRCC in Rome directly since they have actual capacity to intervene. Once the MRCC started assigning rescues to the Libyan Coast Guard, we see more calls going directly to Alarm Phone.
Broadcast Warnings	National Geospatial-Intelligence Agency	2014-01 – 2020-04	1,043	Maritime alerts about ships in distress, often with information about the number of people on board and whether a ship is sinking, disabled, etc. They also contain information on activities like naval operations, hazards, etc.	Only includes publicly issued requests for help; the MRCC seems to assign and coordinate some rescues behind the scenes without producing a Broadcast Warning. There is no formal information on whether an incident involves migrants or not; we estimate this based on the content of the report, keeping reports that we believe are migrant-related. Sometimes warnings reference multiple boats and positions; these have been divided into multiple distinct incidents when possible.
Médecins sans Frontières	Médecins sans Frontières	2015-05 – 2019-12	444	Records of rescues conducted by MSF ships, including information on weather conditions and whether a rescue was initiated by the MRCC or the boat was detected by MSF or another vessel. Records are grouped by operations, each of which may include several rescues.	Only covers the operations of MSF boats; rescues only occur when MSF boats have been deployed in the region.
Missing Migrants Project	IOM	2014-02 – 2019-12	344	Database of incidents in which migrants died or went missing, compiled from media sources, national authorities, etc.	Does not include incidents where everyone survived. Sources are mixed in quality (though the dataset does provide a source quality estimate), and sometimes multiple incidents appear to be reported as one.

3.2 Comparison of alternative data sources

The analysis below is one of the first to compare multiple incident-level data sources, which we summarize in more detail in Table S3.1 of this appendix. We argue that comparing multiple data sources is important because each dataset captures different phenomena. This is illustrated in Fig S3.1 of this appendix, which summarizes the distribution of observed incidents by dataset for the years 2014 - 2019.

The left panel of the figure shows the spatial distribution of all geotagged incidents in each dataset (restricted to incidents which occurred in the Italian, Maltese, or Libyan SAR zones). In the Frontex dataset, location information is unavailable for all incidents before November 2014 or after October 2017 and for incidents within Frontex's operational areas (which are primarily off the European coasts), so most geotagged incidents are clustered near Libya or in the sea between Egypt and Italy. We can see that the MSF and Broadcast Warnings incidents are also largely clustered off the coast of Libya, where the bulk of rescues occurred in 2016 - 2017. In contrast, the Watch The Med data and the IOM Missing Migrants data are more scattered, likely because these datasets capture incidents that may have "fallen through the cracks" of the standard rescue response, because they continue to capture recent incidents, and/or because their associated geocoordinates may be less accurate.[4]

From the right panel of Fig S3.1 of this appendix, we can also see that the coverage of the datasets varies. Frontex reports a peak of over 200 incidents a month, whereas the Watch The Med dataset never contains more than 20 incidents per month for this region. It is also evident that with the growing restrictions on NGO activities, the volume of incidents handled by MSF has declined.

Furthermore, we can observe a shift in the distribution of incidents across datasets over time. For example, the Broadcast Warnings dataset recorded a large volume of incidents from 2015 - 2018. However, as the Italian authorities began turning over rescue responsibility for these calls to the Libyan authorities, it appears that some migrants have substituted to an alternative channel when requesting assistance: the volume of Broadcast Warnings has fallen even as Watch The Med incidents (which are often derived from calls to the independent NGO Alarm Phone) have increased. In

[4]Watch The Med's online reports often associate a single set of geocoordinates with multiple different incidents. IOM attempts to recover incident information from public data sources, and it seems they may use approximate coordinates when no precise data is available.

Fig S3.2 of this appendix, we analyze the text of the Broadcast Warnings to illustrate that the decline in calls directly coincided with the rise in referrals to non-Italian rescue authorities.

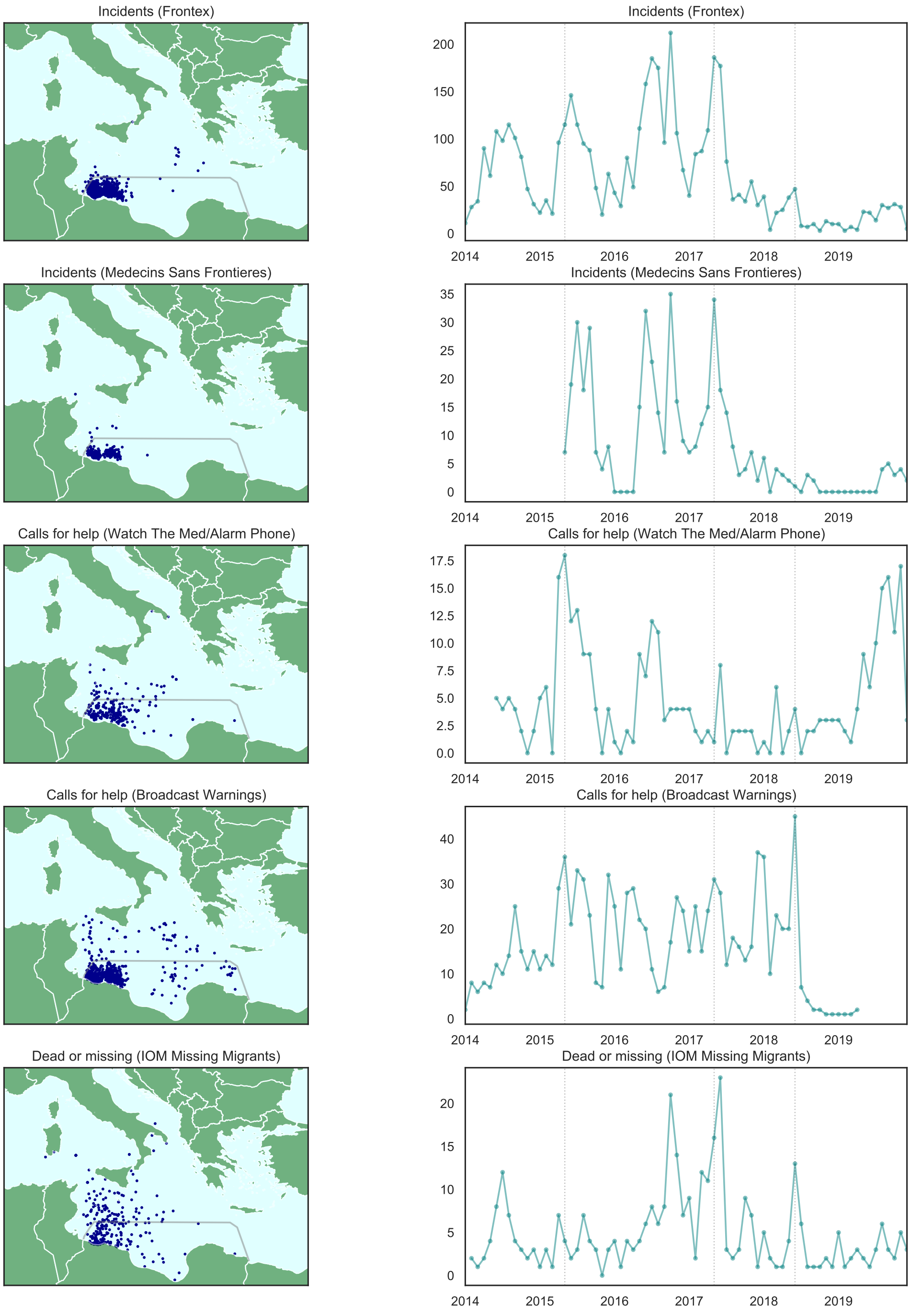

Fig S3.1. The location and frequency of incidents, by dataset. Country borders were obtained from Natural Earth [18]. The linear boundary of Libya's search and rescue zone, shown in grey, was obtained from Watch The Med [19]. The vertical lines in the right panel represent the beginning of Phase 2; the beginning of Phase 3; and the formal recognition of the Libyan SAR zone, respectively.

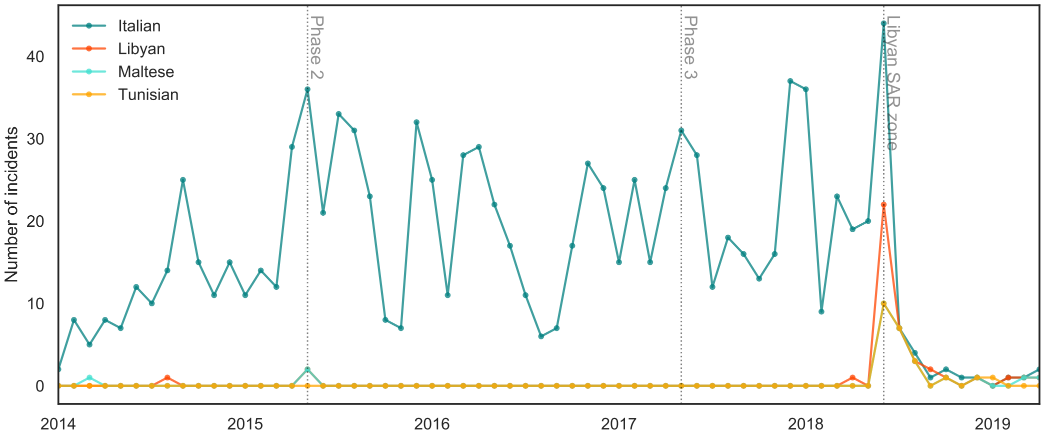

Fig S3.2. The number of incidents by month and rescue authority in the Broadcast Warnings dataset. We identified these incidents by searching the text of the Broadcast Warnings for the phrases "MRCC Rome", "[J]RCC Libya", "Libyan Coast Guard", "RCC Malta", and "MRCC Tunis." Note that a single message might reference multiple rescue authorities, so the categories are not mutually exclusive.

3.3 Descriptive analysis of the incident-level data

3.3.1 Variation in strategic inputs (boat type and size) over time

Using a combination of incident-level data sources, we empirically analyze variations in two key strategic choices made by smugglers: how many migrants to place on each boat, and what type of boat to use. Fig S3.3 of this appendix illustrates the median number of passengers per boat by month for different datasets. As is evident from Fig S3.3a, the median number of people has declined across almost all datasets during our period of study; this decline appears to have accelerated starting around the end of 2017.

If we examine the number of people by boat type, a more nuanced picture emerges. From Fig S3.3c, we can see that prior to mid-2017, the declining number of passengers per boat is driven primarily by the falling size of wooden boats. This may be due to the decreasing availability of large boats, in part because many wooden boats were destroyed by Operation Sophia starting in 2015 [20]. However, it may also result from the fact that rafts became an increasingly viable transport option during this period (since rafts are cheaper, and the growing intensity of rescue efforts made rescue more likely even for less reliable vessels), thus decreasing the relative profitability of launching large wooden boats (which have very high fixed costs). If we examine rubber boats, we can find support for this hypothesis: Fig S3.3b shows that crowding on rubber boats increased through early 2017 while rescue capacity was high, before beginning to decline towards 2018 - 2019. Particularly interesting is the trend for the MSF rescues, which tend to occur within the Libyan SAR zone: we can see that crowding still remains higher for these NGO rescues than for other types of incidents. More generally, the fall in boat sizes is consistent with the notion that smugglers are currently using smaller, less crowded boats to move migrants further out to sea before detection, a hypothesis that we evaluate in more detail below.

Next, we analyze the choice of boats over time, which is illustrated in Fig S3.4 of this appendix. In the Frontex and MSF data shown in Fig S3.4a, we can see that the use of rubber boats peaked in 2016, when NGO rescue activity was at its highest. While MSF continues to rescue a high share of rubber boats, in both datasets we can see an increasing tendency to select wooden boats, which continued through 2019. While the Watch The Med dataset appears to show an increase in the proportion of rubber rafts,

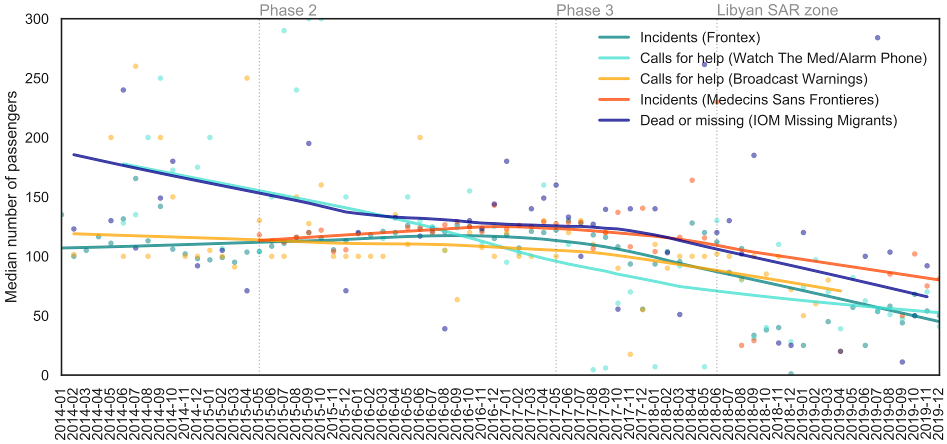

(a) All boats

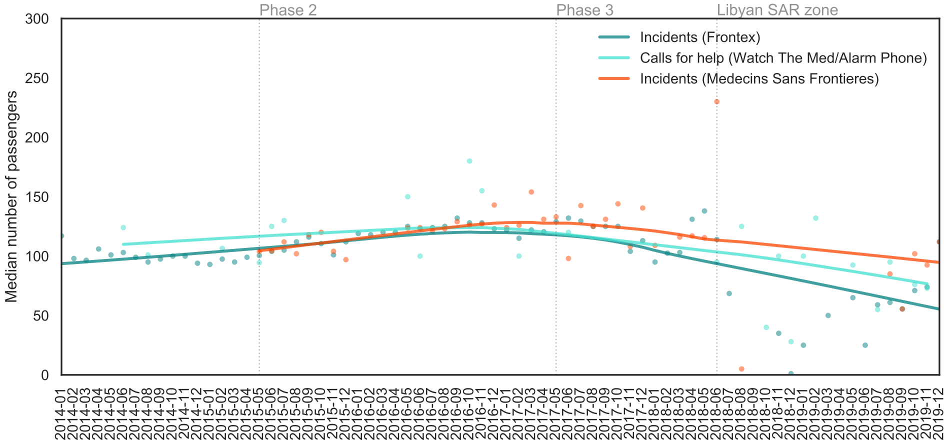

(b) Rubber only

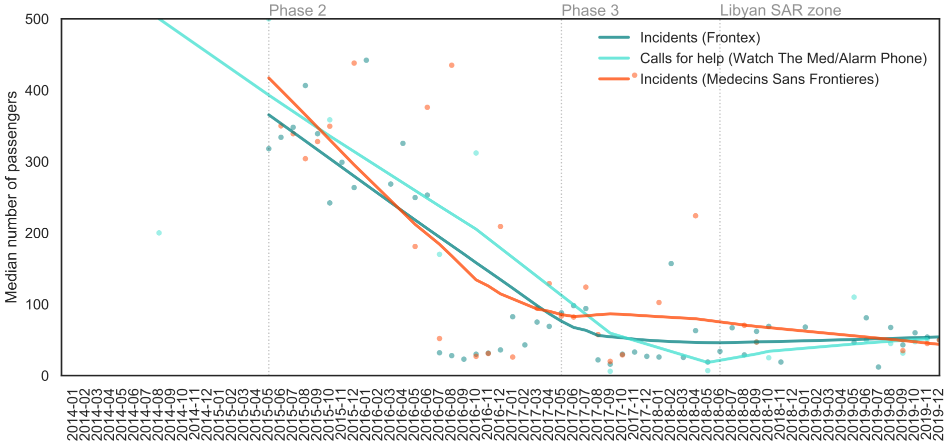

(c) Wooden only

Fig S3.3. The number of people per boat by month and dataset. Each dot represents the median number of passengers per month for the respective dataset, whereas the lines represent locally weighted scatter plot smoothing (LOWESS) fits to the scatter plot trends.

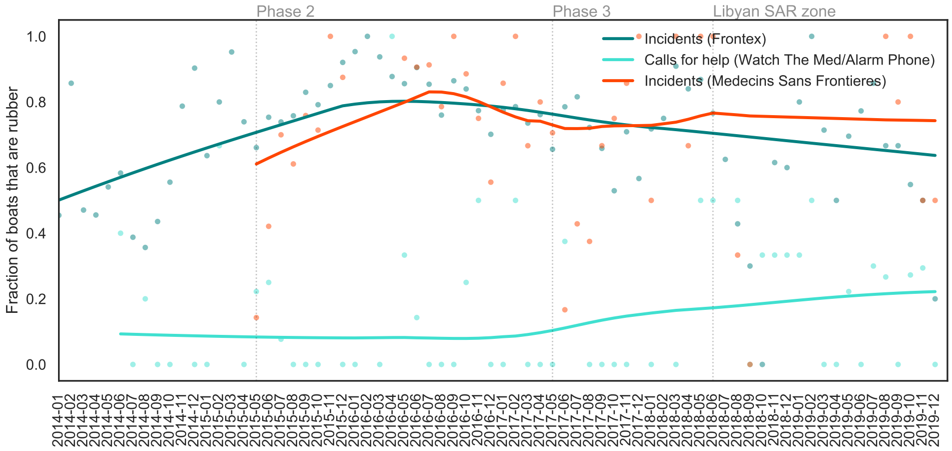

(a) Monthly fraction of rubber boats

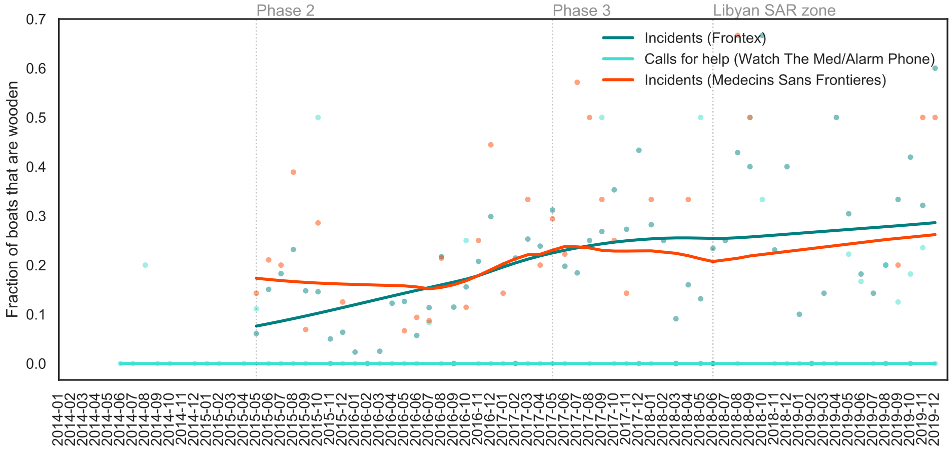

(b) Monthly fraction of wooden boats

Fig S3.4. The choice of boat type by month and dataset. Each dot represents the monthly fraction of rubber or wooden boats for the respective dataset, whereas the lines represent locally weighted scatter plot smoothing (LOWESS) fits to the scatter plot trends. Note: The Frontex and Watch the Med datasets included boat types other than rubber or wooden. Therefore, the trends do not sum to one across both plots.

this may be due to the fact that rubber rafts are increasingly likely to call Watch The Med for help because fewer of them are being independently discovered by NGOs or EU ships near the Libyan Coast, and because in earlier periods these boats had been more likely to call the Italian MRCC for help instead (see Section 3.2 of S3 Appendix for further discussion).

3.3.2 Variation in outcomes (boat location) over time

We have little visibility into the activities of the Libyan Coast Guard, since our incident datasets generally focus on NGO or EU-led rescues. In the absence of comprehensive data on interceptions, we focus on proxy outcomes based on the location of individual incidents.

Boats departing Libya pass from the Libyan SAR zone into EU (Italian/Maltese) SAR zones (these zones are illustrated in Fig 2 of the main paper). In Phase 2, boats in either zone were highly likely to be rescued to Europe. However, starting in Phase 3 boats in the Libyan SAR zone were increasingly likely to be captured and returned to Libya, and crossing into the EU SAR zones was more and more important for securing rescue to Europe. Therefore, the average location of individual incidents in Phase 3 likely reflects how much effort smugglers are investing to avoid detection and/or distress incidents near the Libyan shore.

Fig S3.5 of this appendix plots the median distance to the SAR zone border over time, where negative distances represent incidents on the Libyan side of the border, and positive distances represent incidents on the EU side of the border. We can see that for almost all periods and datasets, the median incident occurs on the Libyan side of the border. Recorded incidents were closest to Libya in Phase 2 when NGO rescues were high, but have gradually moved towards the EU SAR zones as interceptions have grown.

The fact that migrant boats are traveling farther out to sea before being rescued and/or identified by one of these data-collecting actors is likely a result of two different phenomena. On the one hand, a growing share of incidents near the shore are likely filtered out of our datasets by LCG interceptions. On the other hand, as the presence of rescue boats near the Libyan coast has declined, smugglers are aware that boats must go farther to increase their probability of rescue, and may adapt their strategies accordingly. As noted above, they appear to be launching smaller boats and shifting to wooden

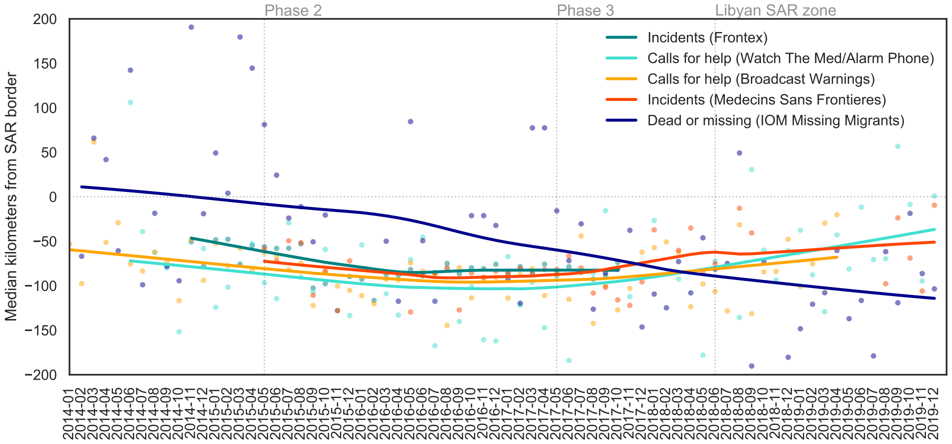

Fig S3.5. Distance from the Libyan SAR zone border - Incident location by month and dataset. Each dot represents the monthly median distance or fraction of incidents for the respective data set, whereas the lines represent locally weighted scatter plot smoothing (LOWESS) fits to the scatter plot trends. In Panel (a), negative distances represent incidents on the Libyan side of the border, and positive distances represent incidents on the EU side of the border. In Panel (b), "land" is defined as the land mass of any country bordering the Mediterranean. In Panels (a) and (b), we note that the Frontex data set only included location data for a subset of time periods and for incidents that occurred *outside* the Frontex operational area, that is, nearer to the coast of Libya.

boats, but migrants on board may also be delaying their decision to call for help until they are farther away from the Libyan coast. Weak evidence for the latter hypothesis may be seen by comparing incident records from Watch The Med/Alarm Phone to the other incident datasets; in recent periods, calls to Watch The Med/Alarm Phone appear to show a slightly larger shift away from land and towards the EU SAR zones.

The one exception to these trends is the IOM missing migrants dataset, in which incidents appear to be moving closer to shore on average. It is important to note that this dataset *does* include deaths recorded off the coast of Libya (for example, IOM reports data on the number of dead and missing people from Libyan interceptions when available, and also reports incidents where bodies wash up on shore) and therefore might contain a more representative sample of incidents in the Libyan SAR zone when interception rates are high. Deaths may be occurring increasingly near the coast for two reasons. First, LCG rescues may be more dangerous for migrants on average, due both to the lack of professionalism and expertise by the LCG, and to the fact that migrants sometimes conduct risky maneuvers to avoid capture by the LCG. Second, it is also likely that a larger proportion of boats near the coast go undetected due to the decrease

in NGO patrol presence, the slower response time of the LCG, and migrants' reluctance to call for help; this may lead to a growing number of sinking incidents in which bodies wash up on shore.

3.3.3 The connection between strategic inputs and outcomes

Finally, we provide summary details on the connection between boat type, crowding, and incident outcomes. We assume that in Phases 1 and 2, reaching a European SAR zone or the Frontex operational area is relatively unimportant to migrants because almost all boats are rescued to Europe regardless of whether or not they are in the Libyan SAR zone. In Phase 3, however, we assume that migrants make the most effort to move away from the Libyan coast before being detected, since exiting the Libyan SAR zone and approaching Europe will make it more likely that they are rescued rather than returned to Libya. At the same time, we expect that the growing rate of interceptions in this period may make the payoffs to different boat sizes and types more pronounced, since a poor choice of boat size or type will leave migrants more vulnerable to interception and/or reliance on help from the LCG in case of distress.

This is consistent with Fig S3.6 of this appendix, which shows increasingly divergent outcomes by boat size and type in Phase 3. From Fig S3.6a, we can see that smaller boats appear to have an advantage in reaching the Frontex operational area. This advantage is most pronounced for boats with less than 50 passengers, followed by boats with 50-100 passengers. From Fig S3.6b, we see that wooden boats are most successful in reaching the Frontex operational area; this is likely because they are more seaworthy in general, although we do see some rubber boats that are able to cross over successfully.[5]

3.4 Inferring smuggler strategy from incident-level datasets

In this section, we have analyzed smuggler strategy from two different angles. First, we have shown that the inputs chosen by smugglers (i.e., the size and type of boat) have varied over time. We also documented a corresponding shift in smuggling outcomes (i.e.,

[5]The Frontex dataset also includes a number of "other" boat types including: fishing boats, motor boats, fiberglass boats, sailboats, etc. On average, these "other" boats are more successful in reaching the Frontex operational area, but we do not analyze them because these boat types can be very heterogeneous, and because they represent a smaller portion (11%) of incidents, relative to rubber boats (74%) and wooden boats (14%).

the location of incidents involving migrants) and shown a correlation between these inputs and outcomes. This provides support for our main strategic model of boat size choice, which has been fit using incident-level data from Frontex.

We conclude with a note on the incident-level datasets analyzed above. Our analysis relies heavily on the Frontex dataset because it is the most comprehensive incident-level data source; we estimate that the number of people in the Frontex dataset closely corresponds to the number of arrivals to Europe reported by IOM (see Section 4.1 of S4 Appendix for details). However, this dataset has a number of limitations, including that it is not publicly available; that it does not contain a full set of exact incident locations; and that, to the best of our knowledge, it does not cover interceptions by the Libyan Coast Guard. We also note that incident records from 2020 were not released for security reasons.

We analyze other incident-level datasets to address these shortcomings, comparing multiple datasets to demonstrate that incidents' average distances to shore are increasing and that smugglers are shifting towards the use of smaller boats and wooden boats. However, we note that each of these datasets records different incident attributes, and each demonstrates different biases and shortcomings. Furthermore, for several of the datasets, the criteria by which incidents were identified or excluded is not entirely clear. Collecting data from multiple alternative sources also required substantial manual effort, and matching incidents across sources was challenging due to variations in the reported location, date, and number of people involved. Likely for this reason, we are unaware of other research that compares incident-level data from multiple different data sources in the region. Finally, we note that across all of these datasets, there is remarkably little transparency into recent activities off the coast of Libya, such as LCG interceptions and aerial surveillance flights run by European authorities to identify migrant boats that have not yet exited the Libyan SAR zone.

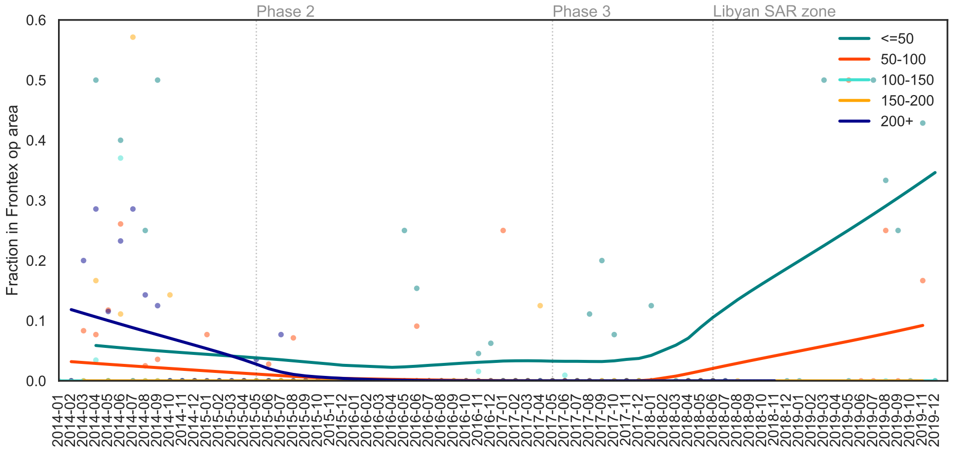

(a) The probability that an incident is in the Frontex operational area, by number of people

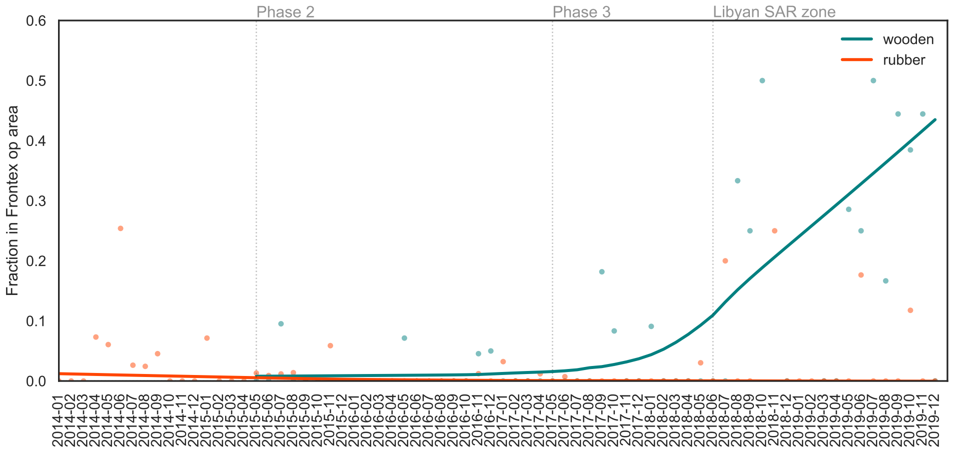

(b) The probability that an incident is in the Frontex operational area, by boat type

Fig S3.6. Incident location by month and dataset, by number of people and boat type. Each dot represents the monthly fraction of incidents in the Frontex operational area for the respective boat size or type, whereas the lines represent locally weighted scatter plot smoothing (LOWESS) fits to the scatter plot trends.

S4 Supplementary details for the incident-level analysis

4.1 Justification of assumptions

Below, we justify the assumptions used in the primary incident-level analysis.

Assumption 1: *Frontex's recorded incidents do not cover interceptions. However, Frontex data is a representative and nearly comprehensive sample of rescue incidents in the region.*

To analyze the representativeness of Frontex's dataset, we compare the number of people involved in Frontex incidents to the total number of sea arrivals to Italy or Malta reported by the IOM. As shown in Fig S4.1 of this appendix, while the datasets do not match exactly, the Frontex dataset appears to capture most people who reached Europe on this route during our study period. A discrepancy emerges in 2018, when the IOM data begins to incorporate data on arrivals to Malta as well as Italy; however, the coverage of the Frontex dataset remains around 70-80%. Therefore, it seems reasonable to assume that the distribution of departure countries in the Frontex dataset approximates the distribution of departure countries in the IOM arrivals data.

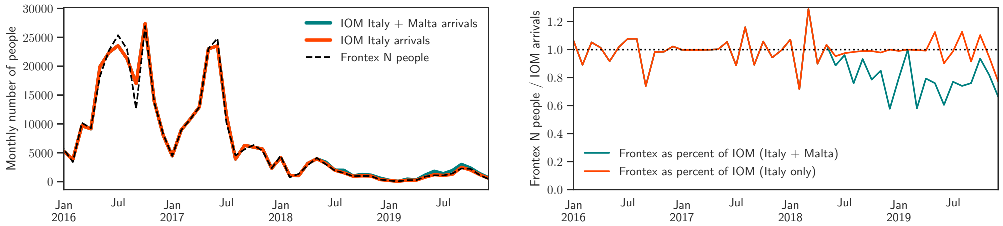

Fig S4.1. Comparison between people involved in Frontex incidents and total arrivals reported by IOM.

Assumption 2: *The smugglers' decision focuses on the probability of interception and does not independently weigh the probability of sinking, which is small.*

In the Frontex incident dataset, only 5 of the 4,365 incidents involving boats departing Libya suffered the loss of all people on board, and the overall fatality rate is 0.2 percent. According to the IOM flows dataset, approximately 2% of all migrants attempting the crossing between 2016 - 2019 have gone dead or missing.

In Section 4.3 of S4 Appendix, we show that Frontex incidents (i.e., incidents in

which a boat was rescued to Europe) were slightly less likely to involve dead or missing migrants in Phase 3, even as smaller boats were used and the incidents appear to have occurred farther from Libya. Therefore, it appears that the strategic changes have not increased the risk of death for passengers, conditional on boats being rescued to Europe.

In the IOM flows dataset, there appears to have been a rise in the fatality rate during Phase 3, but IOM does not report whether these fatalities are associated with the Libyan or the Tunisian route. On the Libyan route, we estimate that fatalities may be closely associated with the probability of interception, because (1) migrants may be exposed to risks in the course of LCG operations; (2) the LCG may have a slower overall response time to distress incidents when it is charged with a rescue; and (3) the efforts of migrants to evade the LCG may lead them to sink without detection. Therefore, we expect that the probability of interception may act as a proxy for the risk of sinking.

4.2 Additional details on the Frontex incident dataset

Below, we briefly provide additional summary statistics on the Frontex incident dataset. In Fig S4.2 of this appendix, we plot the empirical distribution of boat sizes in the dataset. We see that most wooden boats tend to be small, but that there is a long tail of extremely large boats. In contrast, rubber boats generally hold under 200 people, with a peak around 100 - 150 passengers. For this reason, we have focused our estimation on rubber boats. The right panel of the figure illustrates that for both types of boats, the average number of people on board is positively correlated with the quarterly probability of rescue, which is consistent with the hypothesis that smugglers are responding strategically to LCG interceptions by changing the size of the boats they launch.

Table S4.1 of this appendix shows the quarterly number of Libyan incidents in the Frontex dataset by boat type and size. In general, there are more incidents involving rubber than wooden boats, which further motivates our decision to focus on rubber boats. We also note that there are far fewer incidents starting in 2017Q3; this is consistent with the fact that the overall volume of departures fell dramatically once the rate of LCG interceptions increased, and with the fact that an increasing number of boats were presumably intercepted by the LCG before they could be captured in the Frontex dataset.

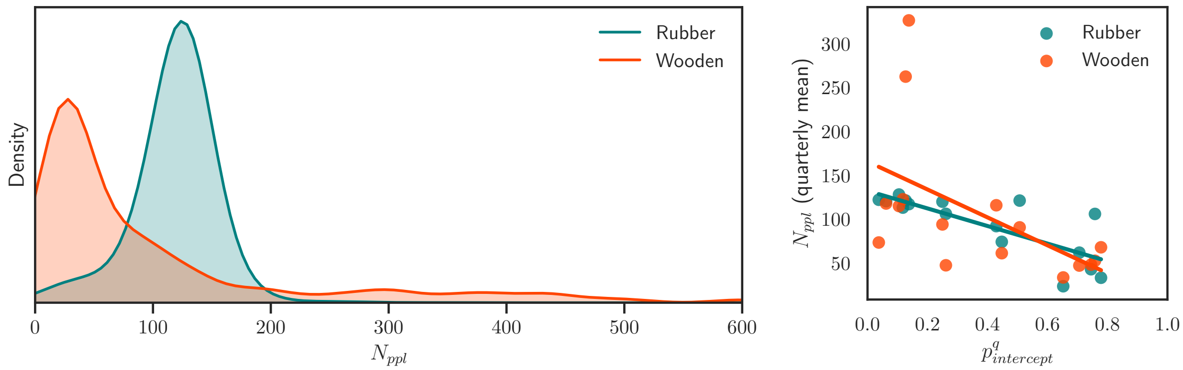

Fig S4.2. The distribution of boat sizes in the dataset (left) and the relationship between average boat size and the quarterly probability of interception (right). Note that the long tail on the distribution of boat sizes extends beyond 600, but the axis range has been limited for clarity of presentation.

Table S4.1. The number of Libyan incidents in the Frontex dataset, by boat type and size.

	Rubber			Wooden			Total
	0<N<= 50	50<N<=100	100<N<=900	0<N<= 50	50<N<=100	100<N<=900	
2016Q1	0	16	118	0	0	3	137
2016Q2	9	15	243	6	1	21	295
2016Q3	16	45	308	58	1	11	439
2016Q4	20	30	250	51	4	20	375
2017Q1	12	22	120	21	12	14	201
2017Q2	6	48	278	32	37	41	442
2017Q3	7	11	92	17	6	7	140
2017Q4	5	17	42	33	3	4	104
2018Q1	5	13	17	8	1	5	49
2018Q2	4	8	33	7	3	3	58
2018Q3	0	0	1	1	2	0	4
2018Q4	3	0	0	2	1	0	6
2019Q1	3	0	0	0	1	0	4
2019Q2	4	4	0	2	2	0	12
2019Q3	5	6	1	3	2	0	17
2019Q4	3	8	3	7	2	1	24
Total	102	243	1506	248	78	130	2307

4.3 T-tests supporting strategic shifts in the Frontex dataset

Next, we briefly compare the characteristics of Frontex incidents originating in Libya during Phase 2 and 3 using two-sample t-tests with unequal variances. This analysis is intended to support the descriptive plots included in Section 3.3 of S3 Appendix. From Table S4.2 of this appendix, we can see that incidents in Phase 3 have a significantly lower average number of people per boat; are significantly less likely to involve rubber boats; and are significantly more likely to occur in the Frontex operational area. Incidents in Phase 3 are less likely to involve dead or missing migrants, and have fewer deaths on average. However, this latter result may be a function of boat size; when we test for differences in the average *proportion* of dead or missing people per boat, we find no significant difference.

Table S4.2. T-tests of strategic shifts in the Frontex dataset.

Variable	Phase 2 Mean	Phase 3 Mean	Difference	P-value
Number of people per transport means	134.857	108.978	25.878***	0.000
Boat type = rubber (vs. wooden)	.844	.731	.113***	0.000
In Frontex operational area	.009	.052	-.043***	0.000
Incident involved dead or missing	.053	.039	.014**	.0400
Number of dead or missing	.331	.187	.144*	.0899
Fraction of dead or missing	.004	.004	-.001	.3196

Phase 2 has been defined as May 2015 - April 2017, whereas Phase 3 has been defined as May 2017 - December 2019.

4.4 Robustness to alternative weighting schemes

To estimate the utility function using incident-level data, we used frequency weights in which each incident was weighted by the total number of incidents in its quarter: $w = \frac{1}{N^q}$. This ensured that each quarter was given equal weight in the estimation and effectively up-weighted incidents from later quarters, when there were fewer incidents observed.

To show that our results are not an artefact of the weighting scheme, in Columns (4)-(6) of Table S4.3 in this appendix we compare the frequency weights with two alternative weighting schemes. Column (4) shows unweighted estimates, whereas Column (5) shows estimates weighted according to the probability of rescue: $w = \frac{1}{p^q_{rescue}}$. The motivation for weighting according to the probability of rescue is that when the probability of rescue is low, each observed incident should be up-weighted because it represents other, unobserved incidents which were filtered out of the dataset by interceptions. However, this weighting scheme is least preferred since it will exacerbate any biases between observed and unobserved incidents that are driven by the rate of LCG interceptions.

When we compare estimates from these alternative weights to the frequency weights in Column (6), we see that the frequency weights lead to estimates that are more extreme in magnitude. That is, the baseline payoff to crowding (α_n) is higher, whereas the penalty to interception (β_n) is more negative. This is unsurprising because, as noted above, the frequency weights place higher weights on incidents later in the dataset, when the probability of interception is higher and the strategic response is more evident.

In Columns (1)-(3) of Table S4.3, we also present the results of a simple model where utility consists only of the first (α_n) term (i.e., the payoff to crowding) and

Table S4.3. Results from the conditional logit model, under different weighting schemes.

			p_{in}			
	(1)	(2)	(3)	(4)	(5)	(6)
$\alpha_{\ 50-100}$	0.868***	0.683***	-0.166	1.225***	1.340***	1.786***
	(0.118)	(0.134)	(0.270)	(0.177)	(0.198)	(0.413)
$\alpha_{\ 100+}$	2.692***	2.373***	0.886***	3.537***	3.704***	3.849***
	(0.102)	(0.117)	(0.264)	(0.158)	(0.176)	(0.604)
$\beta_{\ 50-100}$				-1.740***	-2.178***	-3.587***
				(0.609)	(0.676)	(0.955)
$\beta_{\ 100+}$				-5.161***	-5.986***	-6.511***
				(0.572)	(0.587)	(1.998)
weights	-	rescue	frequency	-	rescue	frequency
pseudo R2	0.459	0.397	0.106	0.483	0.458	0.268
N Obs.	5,553	5,553	5,553	5,553	5,553	5,553

Standard errors in parentheses

* $p < 0.10$, ** $p < 0.05$, *** $p < 0.01$

ignores the penalty to interceptions (β); again, we consider all three weighting schemes. We can see that in this model, estimates of α_n are more conservative in magnitude than in the full model; this may be because the simpler model fails to independently capture the interception penalty associated with larger boats, and this most likely dampens the estimates of α_n.

4.5 Evidence on monetary transfers from migrants to smugglers

In our incident-level analysis, we chose not to explicitly model the revenue, costs, and profits of smugglers. To the best of our knowledge, obtaining consistent enough information to estimate fees for the crossing at given points in time is infeasible. Table S4.4 summarizes different descriptions of the monetary payments from migrants to smugglers, which implies high variation in the amount of the payments according to a range of factors including nationality and boat type.

Table S4.4. Examples of how smuggling fees are described in the literature.

"Criminal networks smuggling migrants to Italy by boat ... in 2017 ... [charged] EUR 1,300 per person. In mid-2018, the smuggling fees increased to EUR 1,800 per person ... " [21]
"Unscrupulous Libyan smugglers, taking advantage of their desperation, charge anywhere from $750 to $3,500 apiece for a place on a boat they say is headed to Italy." [22]
"Prices may range between USD 500 and 2,000, depending on a variety of factors including: nationality, ... ability to drive a boat ... previous support to the smuggler ... need to fill up the boats ... weather conditions ... " [23]
"They are charged approximately LYD 1,200 – 1,500 (USD 200 – 250) for both accommodation and the sea crossing itself. Rubber dinghies bought at the local market are the standard form of transportation but if these are not available or have become more expensive then smugglers might increase ticket prices." [23]
"In terms of the price paid by migrants to be smuggled from Libya to Italy, the amount varies depending of the specific deal agreed between the migrant and the smuggler. However ... in some cases the average price paid by certain nationalities, namely migrants from Horn of Africa countries, has increased from 500 – 800 USD, which was the regular price in previous months, to 1,500 – 2,300 USD to travel aboard a rubber boat. " [24]
" ... information collected from migrants and contacts in Zuwara, Tripoli, Garabulli and Bani Walid since May 2018 shows that the price of the sea crossing was consistently above LYD 1,500 (around €300, at black-market exchange rates at the time of writing) ... with the average price hovering around the LYD 2,300 – LYD 2,500 mark (€460 – €500). This represents a significant increase from the same period in 2017, when the price had dropped to below LYD 1,000, when the black-market exchange rate meant this sum was the equivalent of about €100. There have also been increasing variations in price, in some cases reaching LYD 6,000 – LYD 7,500 (around €1,200 – €1,500)." [25]
"The smuggling fees depend on the services included in the package, but also on the migrants' citizenship, sex and age. Along the different Central Mediterranean subroutes, smugglers tailor the prices to the - real or perceived - economic means of the migrants. Syrians, who are usually wealthier than African migrants, pay more for safer journeys. Negotiations are possible and the prices may vary significantly. One study reported that migrants from sub-Saharan Africa would pay around US$1,000 to be smuggled below deck on a boat from Libya to Europe, whereas a Syrian would pay $2,500 or more for a safer seat. In February 2015, new arrivals to Italy from Libya reported having paid smugglers between US$700 and 1,000 per person to undertake the journey. Some reported having paid as little as $400 for the journey to Lampedusa." [26]
"Prior to 2014, journeys to North Africa and from North Africa to Europe could be categorized according to standard prices. In 2014, however, the price of a journey facilitated by a smuggler was dependent upon the nationality of the migrant (Syrian refugees paid higher prices than sub-Saharan African migrants and asylum seekers), the smuggling ring the migrant came into contact with in Libya, and the level of service that the migrant was willing to pay for (at a higher price, a migrant could secure a place on the top deck of the vessel and receive a life jacket)." [27]
"It is difficult to determine whether significant fluctuations in smuggling fees have occurred along West African, North African and Central Mediterranean routes since 2014. While key informants generally assumed an increase in smuggling fees due to the higher risks and border controls, smuggling fees reported by interviewed migrants show a slight decrease. The sea crossing is almost always paid separately from the rest of the journey, and sometimes paid to different actors. The field research for the Observatory suggests that smuggling fees requested for the sea crossing may have decreased since 2015, when the average was reported at around USD 1,000 for a Sub-Saharan African client ... As smuggling packages vary, and the smuggling fee paid by migrants represents just one of the revenue-generating activities linked to smuggling, the assessment of price variations over time, and of smugglers' profits, is a challenging exercise." [28]
"Smuggling fees varied widely and were almost invariably negotiated. It was common to find two migrants who, having travelled on the same boat, had paid significantly different amounts." [29]
"On Libya's coast, for example, the price for a place on a smuggler's boat has fallen consistently between 2013 and the present. Up to early 2014, it hovered between $1,000 and $1,500 but then began to slide sharply as rescue operations meant smugglers could shift their business model from genuine attempts at crossing the Mediterranean to Italy to merely trying to get to the rescue zone. A seat on a boat from Libya now costs $200 or less, with migrants offered free passage if they bring four or more paying friends." [30]

References

1. UN Treaty Collection. Convention Relating to the Status of Refugees; 1951. https://treaties.un.org/doc/Treaties/1954/04/19540422%2000-23%20AM/Ch_V_2p.pdf.
2. UN Treaty Collection. International Convention for the Safety of Life at Sea; 1974. https://treaties.un.org/doc/Publication/UNTS/Volume%201184/volume-1184-I-18961-English.pdf.
3. UNHCR. Rescue at Sea, Stowaways and Maritime Interception, 2nd Ed.; 2011.
4. UN Treaty Collection. International Convention on Maritime Search and Rescue; 1979. https://treaties.un.org/doc/Publication/UNTS/Volume%201405/volume-1405-I-23489-English.pdf.
5. Shepherd B. The Gravity Model of International Trade: A User Guide (An Updated Version). UN Economic and Social Commission for Asia and the Pacific; 2016.
6. Silva JS, Tenreyro S. The Log of Gravity. The Review of Economics and Statistics. 2006;88(4):641–658.
7. UNHCR. Europe - Refugee and Migrant Arrivals and Dead and Missing Data; 2019. https://data2.unhcr.org/en/documents/details/58460.
8. IOM. Data on Attempted Crossings of the Mediterranean Sea 2016-2019; 2019.
9. US International Trade Commission. Dynamic Gravity Dataset 1948-2016; 2018.
10. Eurostat. Population on 1 January by Age Group, Sex, and Country of Birth; 2023. https://ec.europa.eu/eurostat/databrowser/view/MIGR_POP3CTB/default/table?lang=e
11. The Fund for Peace. Fragile States Index; 2023. https://fragilestatesindex.org/.
12. Box-Steffensmeier JM, Freeman JR, Hitt MP, Pevehouse JCW. Time Series Analysis for the Social Sciences. Analytical Methods for Social Research. Cambridge: Cambridge University Press; 2014.
13. European Council. EU-Turkey statement. 2016.
14. Watch the Med. Reports; 2020. http://watchthemed.net/index.php/reports.
15. National Geospatial-Intelligence Agency. Broadcast Warnings; 2018. https://msi.nga.mil/NavWarnings.
16. MSF. Search and Rescue - Interactive Map; 2020. http://searchandrescue.msf.org/map.html.
17. IOM. Missing Migrants Project; 2021. https://missingmigrants.iom.int/downloads.
18. Natural Earth. 1:10m Cultural Vectors; 2020.
19. Watch the Med. Search and Rescue Zone; 2020. https://watchthemed.net/json/layer/9.
20. UK House of Lords, European Union Committee. Operation Sophia, the EU's Naval Mission in the Mediterranean: An Impossible Challenge; 2016. 14th Report of Session 2015–16.

21. Frontex. After the Money: Prices for People Smuggling on Central and Western Mediterranean Routes; 2020. https://frontex.europa.eu/media-centre/news/news-release/after-the-money-prices-for-people-smuggling-on-central-and-western-mediterranean-routes-EHDfJg.

22. Baker A. Rescue at Sea - A Week on Board a Refugee Recovery Ship. TIME. 2016.

23. UNHCR, Altai Consulting. Mixed Migration Trends in Libya: Changing Dynamics and Protection Challenges; 2017.

24. Frontex. JO EPN Triton 2016 - Biweekly Analytical Report; 2016.

25. Micallef M. The Human Conveyor Belt: Trends in Human Trafficking and Smuggling in Post-Revolution Libya. Geneva, Switzerland: The Global Initiative Against Transnational Organized Crime; 2017.

26. UNODC. Global Study on Smuggling of Migrants. Vienna, Austria; 2018.

27. Malakooti A. The Dynamics of Migrant Smuggling in North Africa: Focus on the Central Mediterranean Route. IEMed Mediterranean Yearbook. 2016; p. 5.

28. UNODC Observatory on Smuggling of Migrants. Key Findings on the Characteristics of Migrant Smuggling in West Africa, North Africa and the Central Mediterranean; 2021.

29. Sanchez G. Migrant Smuggling in the Libyan Context: Re-Examining the Evidence. In: Migration in West and North Africa and across the Mediterranean: Trends, Risks, Development and Governance. Berlin: IOM GMDAC; 2020.

30. Reitano T. What Pricing Tells Us About the Nature of the Smuggling Business. The New Humanitarian. 2017.
